# Nanobody Immunolabelling and three-dimensional imaging reveals spatially restricted LYVE1 expression by kidney lymphatic vessels in mice

**DOI:** 10.1186/s12951-025-03759-3

**Published:** 2025-10-13

**Authors:** Eva Maria Funk, Daniyal J. Jafree, Nils Rouven Hansmeier, Clàudia Abad Baucells, Rose Yinghan Behncke, Gideon Pomeranz, Maria Kolatsi-Joannou, William J. Mason, Dale Moulding, Lauren G. Russell, Ayshwarya Subramanian, Sascha Ulferts, Laura Wilson, David A. Long, René Hägerling

**Affiliations:** 1 Research Group ’Lymphovascular Medicine and Translational 3D-Histopathology’, Institute of Medical and Human Genetics, Charité - Universitätsmedizin Berlin, Berlin, Germany; 2 Berlin Institute of Health at Charité - Universitätsmedizin Berlin, BIH Center for Regenerative Therapies, Berlin, Germany; 3 Berlin Institute of Health at Charité - Universitätsmedizin Berlin, BIH Biomedical Innovation Academy, BIH-MD Stipendium Program, Berlin, Germany; 4 Developmental Biology & Cancer Research & Teaching Department, UCL Great Ormond Street Institute of Child Health, UCL, London, UK; 5 UCL Centre for Kidney and Bladder Health, University College London, London, UK; 6 Wellcome Trust Sanger Institute, Hinxton, UK; 7 Research Group Development and Disease, Max Planck Institute for Molecular Genetics, Berlin, Germany; 8 Department of Nephrology and Medical Intensive Care, Charité Universitätsmedizin Berlin, Berlin, Germany; 9 Department of Molecular Biology and Genetics, College of Arts and Sciences, Cornell University, Ithaca, NY USA; 10 Clinician Scientist Program, Berlin Institute of Health at Charité - Universitätsmedizin Berlin, BIH Academy, Berlin, Germany

**Keywords:** 3D microscopy, Imaging, Kidney, Lymphatic heterogeneity, Nanobodies, Vascular biology

## Abstract

**Background:**

Lymphatic vessels are complex three-dimensional (3D) structures that facilitate tissue fluid clearance and regulate immune responses during health and inflammation. Recent advances in wholemount immunolabelling and 3D imaging have provided insights into organ-specific heterogeneity of lymphatic vessel structure and function. However, the visualisation of lymphatic vessels deep within an intact organ remains a challenge. We hypothesised that nanobodies, single-domain antibodies raised in camelid species, would result in improved labelling of lymphatics in intact mouse organs, without loss of information due to tissue sectioning or inadequate penetration of conventional antibodies into intact tissues.

**Results:**

We generated and characterised nanobody clones targeting lymphatic vessel endothelial hyaluronan receptor 1 (LYVE1), a marker of lymphatic vessels. Compared with a conventional anti-LYVE1 polyclonal antibody, nanobodies were able to penetrate whole mouse organs more rapidly and at a greater depth, facilitating labelling of lymphatic vessel networks within intact mouse organs. Utilising this new tool, we found that lymphatics within the kidney, an organ in which labelling of these vessels is challenging, have spatially restricted LYVE1 expression compared with lymphatics of skin, heart, and lung. The appearance of LYVE1- kidney lymphatics coincided with the early postnatal period in mice, with single-cell RNA sequencing analysis revealing their transcriptome to be enriched for markers of either collecting vessels or lymphatic valves.

**Conclusions:**

Our findings highlight a characteristic feature of kidney lymphatic vessels, whilst providing a novel experimental tool for characterisation, isolation, or perturbation of lymphatic vessels in health and disease.

**Graphical Abstract:**

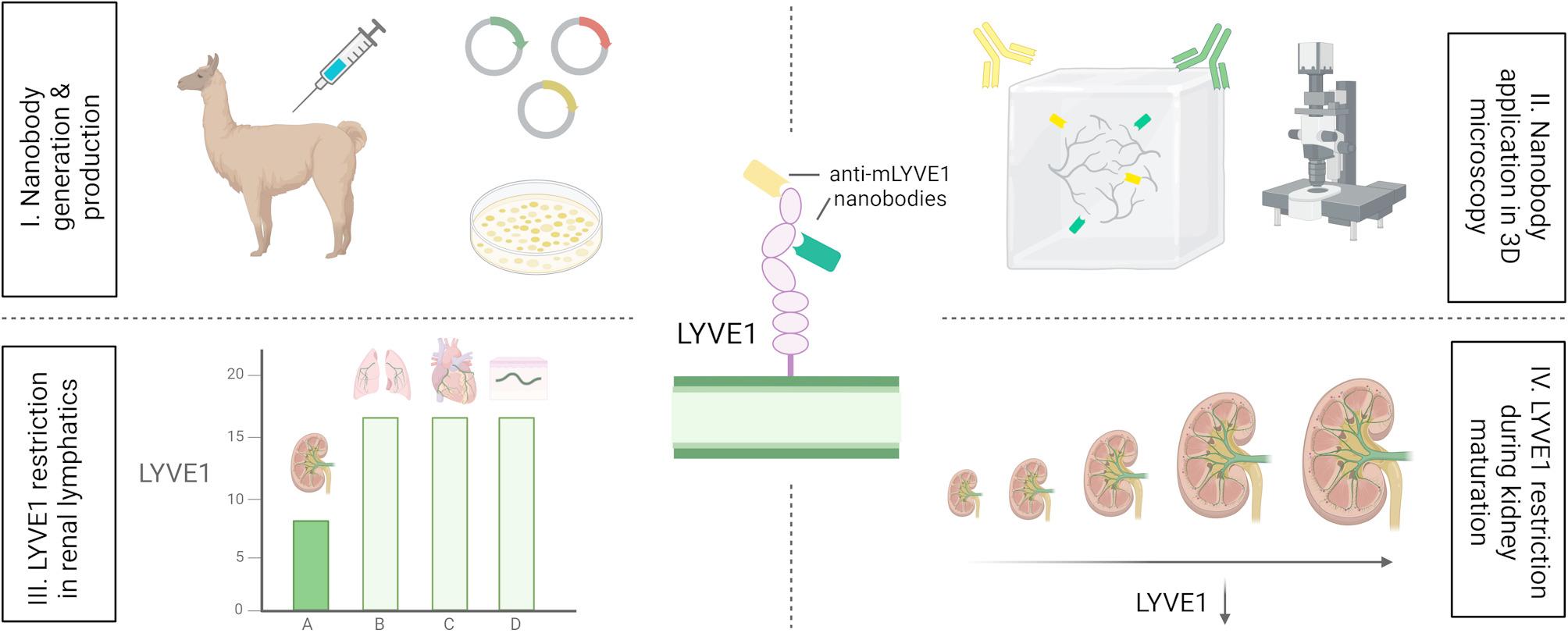

**Supplementary Information:**

The online version contains supplementary material available at 10.1186/s12951-025-03759-3.

## Background

The lymphatic vasculature comprises a complex network of blind-beginning vessels with roles in tissue fluid homeostasis and the clearance of immune cells and macromolecules to modulate inflammatory responses [1]. Lymphatics are present in nearly all adult organs, and have been implicated in a wide range of pathological contexts including cancer metastasis [2] and anti-tumour immunity [3], cardiovascular diseases [4], and autoimmunity [5]. The range of diseases in which lymphatics have been implicated highlight organ-specific heterogeneity in their structure and function [6]. Further evidence for lymphatic vessel heterogeneity comes from their organ-specific roles, including intestinal dietary lipid absorption [7], cerebrospinal fluid clearance [8] and regulation of intraocular drainage [9]. However, due to their complex, three-dimensional (3D) structure, and relative rarity compared to other cell types, traditional histological and imaging techniques fall short in accurately identifying and outlining the structural features of lymphatic vessels. This limitation poses significant challenges in understanding organ-specific heterogeneity of lymphatic vasculature, underscoring the urgent need for improved technologies for lymphatic labelling and imaging [10].

Recently, whole-mount immunofluorescence has enabled the analysis of the complete lymphatic vascular network of mouse organs within which these vessels are superficially located, such as the meninges [11, 12], skin [13, 14], and heart [15, 16]. In certain organs, such as the kidney [17, 18] and liver [19, 20], lymphatics are located deep within the tissue, necessitating optical clearing techniques that homogenise tissue refractive index [21]. This approach has been successfully used to characterise the emergence of lymphatics in mouse embryogenesis [22] and appreciate structural changes to lymphatics in mouse models of kidney disease [23], human lymphedema [24] and transplant rejection [25]. However, without using alternative strategies such as genetic reporter mice [26], lengthy labelling times [27] or specialised perfusion equipment [28], wholemount immunolabelling is limited by the depth of penetration of conventional IgG antibodies into intact organs.

We hypothesised that harnessing camelid-derived single domain antibodies, termed nanobodies, with a small size of approximately 15 kDa, would enable rapid deep tissue penetration, compared with conventional IgG antibodies [29–31], making them ideally suited for immunolabelling lymphatics in intact organs. To this end, we developed nanobodies targeting lymphatic vessel endothelial hyaluronan receptor 1 (LYVE1), a CD44-like transmembrane glycoprotein [32] and widely used lymphatic vessel marker [33]. We validated these anti-LYVE1 nanobodies before utilising them for 3D imaging and quantitative analysis, finding that nanobodies were able to penetrate whole mouse organs more rapidly and at a greater depth than conventional immunolabelling enhancing the visualisation of lymphatic vessels. In so doing, we reveal a novel characteristic feature of lymphatics in the mouse kidney, finding that these vessels have a spatially restricted expression of LYVE1 compared to lymphatics in other organs, a phenomenon which occurs from an early postnatal stage.

## Methods

### Llama immunization and nanobody library construction

For nanobody generation, a recombinant murine LYVE1 protein, consisting of 288 amino acids, fused to a His10 tag, was expressed in human embryonic kidney 293 cells. The recombinant protein was injected alongside Adjuvant P (3111, GERBU Biotechnik GmbH, Heidelberg, Germany) weekly on six consecutive occasions into a Ilama (*Lama glama*). Forty days after the initial immunization, 100 mL of anticoagulated blood were collected from the Ilama, 5 days after the final antigen injection. Peripheral blood lymphocytes were isolated and total RNA extracted for cDNA first-strand synthesis using oligo(dT) primers. Sequences encoding variable nanobodies were subsequently amplified by PCR, digested with the restriction enzyme *SapI*, and cloned into the *SapI* site of the phagemid vector pMECS. Electrocompetent *E. coli* TG1 cells (60502, Lucigen, Middleton, WI, USA) were then transformed with the variable domain of heavy-chain-only antibody (VHH) sequence harbouring pMECS vectors, resulting in a nanobody library comprising 10^9^ independent transformants. This process has previously been described in detail [34].

### Biopanning and identification of anti-mouse nanobodies specific to LYVE1

Phage enrichment and biopanning were carried out as described [34]. Briefly, the previously constructed nanobody library was panned for 3 rounds on solid phase coated with mouse LYVE1 (100 µg/mL in 100 mM NaHCO_3_, pH 8.2) yielding a 400-fold enrichment of antigen-specific phages after the 3rd round of panning. A total of 380 colonies were randomly selected and assayed for mouse LYVE1-specific antigens by ELISA, again using mouse LYVE1 and additionally mouse LYVE1 fused to human IgG1 Fc at the C-terminus (50065-M02H, Sino Biological, Beijing, China). To exclude potential nanobody clones binding to human IgG1 Fc, human IgG1 Fc (10702-HNAH, Sino Biological) was utilized as a control, as well as blocking buffer only (100 mM NaHCO_3_, pH 8.2). After comparing binding specificity of different clones with the control values, 278 colonies were identified as positive for mouse LYVE1 binding. Using the sequence data, the number of possible nanobody candidates was further narrowed down to 98, of which 96 were able to specifically bind both mouse LYVE1-His10 and mouse LYVE1 fused to human IgG1 Fc. The remaining unique clones derived from 21 different B cell lineages according to their complementary determining region (CDR) 3 groups. Considering the different B cell linages and robustness of ELISA screening data, 6 clones were selected for further experiments.

### Cloning of nanobody sequences in expression vector

To generate 6xHis-tagged nanobodies, nanobody sequences were cloned from the pMECS phagemid vector into the pHEN6c expression vector. Initially, nanobody sequences were amplified by PCR using the following primers:

 (1) 5’ GAT GTG CAG CTG CAG GAG TCT GGR GGA GG 3’.

 (2) 5’ CTA GTG CGG CCG CTG AGG AGA CGG TGA CCT GGG T 3’.

PCR products were purified (28104, QIAquick PCR Purification Kit,, Qiagen, Hilden, Germany) and digested for 20 min at 37 °C with *PstI-HF* (R3140, New England Biolabs, Ipswich, MA, USA) and *BstEII-HF* (R3162, New England Biolabs) restriction enzymes, while empty pHEN6c plasmids were concurrently digested with the same restriction enzymes. The empty pHEN6c plasmids, however, were supplemented by 5 units of heat-inactivated (5 min at 80 °C) FastAP™ alkaline phosphatase (EF0651, Thermo Fisher Scientific, Waltham, MA, USA). Digestion products were purified (28104, QIAquick PCR Purification Kit, Qiagen) and subjected to T4 DNA ligase-mediated ligation reactions. The ligation reaction was performed at 16 °C for 16 h using 2.5 units of T4 DNA ligase (M0202, New England Biolabs). Subsequently, newly generated nanobody sequence harbouring pHEN6c plasmids were transformed into WK6 *E. coli* cells (C303006, Thermo Fisher Scientific) and analysed to determine correct nanobody sequence integration by Sanger DNA sequencing using the following primers:

 (1) 5’ TCA CAC AGG AAA CAG CTA TGA C 3’.

 (2) 5’ CGC CAG GGT TTT CCC AGT CAC GAC 3’.

### Production and purification of anti-LYVE1 nanobodies

WK6 *E. coli* carrying pHEN6c-Nanobody plasmid were cultivated at 37 °C, shaking in 1 L `Terrific Broth` medium (2.3 g/L KH_2_PO_4_, 16.4 g/L K_2_HPO_4_-3H_2_O, 12 g/L tryptone, 24 g/L yeast extract, 0.4% (v/v) glycerol) complemented with 100 µg/mL ampicillin, 2 mM MgCl_2_, and 0.1% (w/v) glucose. Nanobody expression was induced at an OD600 of 0.6–0.9 by adding 1 nM isopropyl ß-D-1-thiogalactopyranoside (IPTG). After an incubation period of 16 h, the nanobodies were extracted by centrifugation (8000× g, 8 min, RT). 18 mL of TES/4 buffer (0.05 M Tris [pH 8.0], 0.125 mM EDTA, 0.125 M sucrose) were added for 1 h of shaking on ice. The cell suspension was then centrifuged (8000× g, 30 min, 4 °C), and periplasmic protein-containing supernatant collected. For 6xHis-tagged nanobody extraction, HIS-Select^®^ nickel affinity gel (P6611, Sigma-Aldrich, Darmstadt, Germany) was applied according to the manufacturer’s instructions. The solution was loaded on a PD-10 column (17-0435-01, GE healthcare, Chicago, IL, USA) and nanobodies eluted via 3 × 1 mL 0.5 M imidazole in phosphate-buffered saline (PBS) (I2399, Sigma-Aldrich). An overnight dialysis (3 kDa MWCO, 66382, Thermo Fisher Scientific) against PBS was carried out to remove undesirable imidazole from the nanobody solution.

### Coomassie-blue stained SDS PAGE and Western blotting

To verify nanobody production and pureness, sample protein was separated by molecular weight using established sodium dodecyl sulphate-polyacrylamide gel electrophoresis (SDS-PAGE). For each clone, 5 µg of denatured protein with 0.04% (w/v) OrangeG in ddH_2_O) were loaded onto the gel alongside a pre-stained protein ladder (ab116029, Abcam, Cambridge, UK). For protein visualisation, gels were treated with Coomassie staining solution (0.1% (w/v), Coomassie Brilliant Blue R-250 (1610400, Bio-Rad Laboratories Inc., Hercules, CA, USA), 50% (v/v) methanol, and 10% (v/v) glacial acetic acid in ddH_2_O for 1 h, followed by incubation with Coomassie destaining solution (50% ddH_2_O, 40% methanol, 10% acetic acid (v/v/v)). Alternatively, gels were blotted onto a nitrocellulose membrane (1620112, Bio-Rad Laboratories Inc.) and nanobodies identified using a primary anti-His antibody (12698, Cell Signaling Technology, Danvers, MA, USA) and a secondary anti-rabbit antibody (926-32211, LI-COR Biosciences, Lincoln, NE, USA). Subsequently, blots were analysed by an Odyssey^®^ Fc Imaging System (LI-COR Biosciences).

### Mouse husbandry and acquisition of mouse tissues

C57BL/6 wildtype mice, or *Lyve1*^*Cre − eGFP*^ mice [35] on the C57BL/6 background were utilised in this study. All animal experiments were carried out under a UK Home Office project license (PPL: PP1776587) in compliance with the UK Animals (Scientific Procedures) Act 1986 or approved by German federal authorities (LaGeSo Berlin) under the licence number ZH120. Nutrition and water were available to animals *ad libitum*. Mouse embryos were staged by time-matings, considering embryonic day (E) 0.5 to be the morning a copulation plug was detected. Adult mice or pregnant mice were sacrificed using CO_2_ inhalation and cervical translocation as a Schedule 1 procedure. The desired organs were obtained from embryonic, juvenile, or adult mice, washed in PBS, and subsequently fixed in 4% (w/v) paraformaldehyde (PFA) in PBS for 4 h at 4 °C to preserve tissue integrity. After fixation, samples were thoroughly washed in three changes of PBS and stored at 4 °C in PBS containing 0.03% (w/v) sodium azide until further processing.

### Antibodies for Immunofluorescence

The following commercially available antibodies were used: rabbit monoclonal anti-His antibody (12698, Cell Signaling Technologies) [1:200], donkey polyclonal anti-rabbit IgG Alexa Fluor™ 647 antibody (A31573, Invitrogen, Waltham, MA, USA) [1:1000], donkey polyclonal anti-rabbit IgG Highly-Cross-Absorbed Alexa Fluor™ 647 antibody (A32795, Invitrogen) [1:1000], goat polyclonal anti-mLYVE1 (AF2125, R&D Systems, Minneapolis, MN, USA) [1:100], donkey polyclonal anti-goat IgG Alexa Fluor™ 568 antibody (A11057, Invitrogen) [1:1000], donkey polyclonal anti-goat IgG Highly cross-absorbed Alexa Fluor™ 488 + antibody (A32814, Invitrogen) [1:1000], hamster monoclonal anti-Podoplanin (14-5381-82, Invitrogen) [1:200], goat polyclonal anti-Syrian hamster IgG Cross-Absorbed Alexa Fluor™ 546 antibody (A-21111, Invitrogen) [1:1000], chicken polyclonal anti-GFP (ab13970, Abcam) [1:200], donkey anti-chicken Highly cross-absorbed Alexa Fluor™ 488 + antibody (A32931TR, Invitrogen) [1:1000], rat monoclonal anti-mF4/80 antibody (MCA497G, BioRad) [1:50], donkey anti-rat Highly cross-absorbed Alexa Fluor™ 488 + antibody (A48269, Invitrogen) [1:1000]. Nanobodies were detected by anti-His staining in combination with an Alexa Fluor™ dye-conjugated secondary antibody. An overview of antibody combinations used for each experiment including concentration and incubation time can be found in Table S1.

### Zenon labelling of IgG antibodies

Anti-His antibodies were labelled using the Zenon™ Rabbit IgG Labeling Kit (Z25306, Thermo Fisher Scientific) by incubating the antibody with Component A for 5 min, followed by blocking with Component B for another 5 min. The labelled antibody was used within 30 min.

### Direct labelling of nanobodies

Anti-mouse LYVE1 nanobodies were directly labelled with Alexa Fluor™ 647 NHS-Ester (A20006, Invitrogen) by reacting 1 mg of nanobody with 1 mg of dye in NaHCO₃ buffer (pH 8.0) for 1 h at room temperature. Labelled nanobodies were purified using a NAP™-10 column (Sephadex™ G-25, Thermo Fisher) and stored in PBS with 0.03% (w/v) sodium azide.

### Immunofluorescence staining of cryosections

Snap-frozen 5 μm sections of E14.5 wildtype mice were stained with nanobodies (0.1 µg/mL) and appropriate control antibodies as previously described [36]. Visualisation of representative regions was accomplished using an Axioscope5 fluorescence microscope (Zeiss, Oberkochen, Germany) equipped with a Plan-NEOFLUAR 40x/0.75 objective (Zeiss).

### Wholemount Immunofluorescence staining and optical clearing

Mouse organs were either cut into smaller sections measuring 0.5–2 mm thickness (for studies in Figs. 2A, 3 and 4, Fig. S3B) using a scalpel at room temperature or processed as whole specimens (for studies in Figs. 2B and 4A (E18.5-P5), Fig. S3A, Fig. S4). Tissues were dehydrated using an ascending methanol series and bleached overnight at 4 °C in a solution containing 5% H_2_O_2_ (VWR Chemicals, Radnor, PE, USA) in methanol. Wholemount staining was performed as previously described [22]. For optical clearing, samples were treated with methanol for dehydration followed by BABB (1:2 benzyl alcohol and benzyl benzoate) immersion, as described in [22]. Intestine samples were not optically-cleared, but mounted in fluorescent medium (S3023, Agilent Technologies, Santa Clara, CA, USA). Details on antibodies used, incubation periods and concentrations are outlined in Table S1.

### Wholemount Immunofluorescence staining and optical clearing of embryonic and postnatal day one specimens

Embryonic and early postnatal (P) kidneys were initially dehydrated and bleached as described above. Following rehydration, samples were permeabilized overnight in a 5% solution of 3-((3-cholamidopropyl) dimethylammonio)-1-propanesulfonate in ddH_2_O and blocked in PBS supplemented with 0.2% (v/v) Triton X100, 10% (v/v) DMSO, and 6% (v/v) goat serum. Nanobodies (10 µg/mL) and antibodies were diluted in antibody solution (PBS + 0.2% v/v Tween20 + 0.1% v/v heparin solution + 5% v/v DMSO + 3% v/v goat serum + 0.1% w/v saponin) and incubated for a duration between 4 (nanobodies) and 24 h (IgG antibodies) at 4 °C. Between staining steps, samples were washed in PBS-Tween20. Clearing was performed with BABB as previously described for embryonic kidneys [37].

### Confocal and light sheet imaging

Specimens were imaged using two different microscopy systems. For confocal imaging, an LSM 880 Upright Confocal Multiphoton microscope (Zeiss) equipped with a 20x/NA 1.0 W-plan Apochromat water immersion objective was utilized. A comprehensive description of the setup specific to BABB-cleared specimens for this microscope can be found in previously published work [37]. Intact mouse organs were imaged by LaVision Ultramicroscope II with a LaVison BioTec MVPLAPO 2x OC OBE objective. Various magnifications were employed, and image acquisition utilized a step size of 2 μm. Imaging thresholds were set individually for each channel during image acquisition and post-processing. This approach was chosen to accommodate the variability in autofluorescence and other factors, and to ensure optimal signal detection for each channel.

### Image processing and 3D rendering

2D immunofluorescence images were subjected to post-processing using ZEN 3.4 (blue edition) software from Zeiss. Single channels were extracted and saved in TIFF format. Z-stack datasets were processed and 3D-rendered using Imaris (version 9.8, Oxford Instruments Abingdon, UK). To reduce non-specific background signal, single channels were subjected to the Isosurface Render function. Images of the 3D-rendered data were captured using the Snapshot function and saved in TIFF format. Videos were captured using the Animation function.

### Quantification of signal-to-background ratio

Signal-to-background ratio was quantified using ImageJ 2.24/1.54f (https://github.com/imagej/ImageJ [38]), . Ten intensity values were randomly selected in vessel areas and areas that showed no specific staining. The mean values for signal and background areas were calculated and the signal-to-background ratio was determined using the formula: Signal-to-background ratio = mean signal/mean background.

### Calculation of correlation coefficients

Pearson’s correlation coefficients r were calculated using the Imaris Coloc function. For very large microscopy files, colocalization analysis was performed on representative regions of interest, as full-volume analysis was not computationally feasible on the available hardware. For colocalization of nuclear GFP staining and endothelial nanobody staining, an object-based approach was applied. Spots were segmented independently for GFP and LYVE1 signals using the Imaris Spots function. For each LYVE1 spot, the shortest distance to the nearest GFP spot was determined. LYVE1 spots within 25 μm of a GFP spot were classified as colocalized, and the coefficient was calculated as the proportion of colocalized LYVE1 spots relative to the total number of LYVE1 spots.

### Quantitative analysis of 3D imaging volumes

The quantitative analysis of three-dimensional volumes commenced with the binarization of single channels using Imaris. The binarization process was conducted using the Isosurface Render function, and for E18.5 and P1 samples, 3D cropping was applied to exclude regions with high podoplanin (PDPN) intensity at the kidney surface, thus enhancing binarization accuracy. During surface rendering, the threshold was set to absolute intensity, with manual adjustments to ensure the inclusion of all relevant structures. To eliminate smaller non-specific signals, structures were filtered based on the number of voxels. Following surface generation, PDPN channel-derived non-lymphatic structures, such as glomeruli, were manually removed using the selection function. Subsequently, the channel of interest was masked using specific settings: constant inside/outside, setting voxels outside the surface to 0.00, and inside the surface to the maximum intensity of the prepared channel. All channels except the newly created masked channel were then deleted, and the single channel was saved in TIFF format. With the binarized files prepared, TIFF files were imported into the VesselVio application [39]. The analysis settings were configured as follows: unit µm, resolution type anisotropic with individual sizes of samples, analysis dimensions 3D, image resolution 1.0 µm^3^, and filters applied to isolate segments shorter than 10.0 µm and purge end-point segments shorter than 10.0 µm. Analysis results were automatically saved in Microsoft Excel files by VesselVio. Among the parameters offered by VesselVio, vessel volume was selected as the parameter for further analysis, as it considers both vessel length and width. To account for variability in sample size and volume, vessel volumes (LYVE1 and PDPN) were normalized by dividing the measured vessel volume by the total sample volume, calculated based on the Imaris output for the x, y, and z dimensions. This normalized value was referred to as ‘relative volume’ [40].

### Single-cell RNA sequencing analysis

The generation of the mouse kidney scRNA-seq data used in this study has been described [41]. In brief, a regional enrichment strategy was used to isolate hilum, cortex and medulla separately from 12-week-old C57Bl/6 mouse kidneys (*n =* 14), before single-cell droplet capture using the 10X Genomics platform (10x Genomics, Pleasanton, USA) and sequencing using an Illumina HiSeq X system (San Diego, USA). The count matrix corresponding to the lymphatic cluster was isolated and re-processed, applying a standard workflow using Seurat (v5) in R [42]. The Harmony package [43] was used for integration, using the mouse identifier as a batch variable. Differential expression analysis was performed using the *FindAllMarkers* function, utilising Wilcoxon rank sum tests. Marker genes are presented with average log-fold change (log2FC) values and an adjusted p value of < 0.05 was considered as statistically significant. Gene ontology (GO) analysis was applied using the Panther database [44], using statistical overrepresentation tests and calculating the false discovery rate (FDR) for each GO term.

### Sample size Estimation and statistical analysis

Sample size was estimated based on prior publications of developmental studies of kidney lymphatic vessels [40] and studies investigating lymphatics in adult mice organs [9, 16]. These indicated that 6–8 animals per experiment would be sufficient to power statistical analyses. Statistical analysis was conducted using Prism (v8, GraphPad by Dotmatics, Boston, MA, USA) and RStudio version 12.0 (Posit, Boston, MA, USA). To determine the significance of differences in volume between LYVE1 and PDPN at individual time points and individual organs, a paired student’s *t*-test was carried out. For comparisons of Pearson correlation coefficients r, one-way ANOVA followed by Tukey’s post hoc test was applied. To assess statistical significance across different time points a rank-based approach using the R package nparcomp [45] with the function mctp1 (multiple comparisons for relative contrast effect testing) was chosen. This approach allows for nonparametric multiple comparisons to evaluate relative contrast effects. A p value of less than 0.05 was considered statistically significant for all tests. Quantitative data was visualised using Prism. All graphical representations present individual data points either by region of interest or by animal, along with the mean and standard error of the mean.

### Preparation of figures and videos

The graphical abstract was created in BioRender.com. The figures presented in this paper were prepared using the free software tool Inkscape 1.1.0 (Inkscape Project, 2020) for graphic design and layout. Any modifications made to the images, such as adjustment of brightness and contrast, were applied consistently throughout one panel to maintain uniformity across all coherent single and merged channel images. Videos were edited in Clipchamp (Microsoft, Redmond, WA, USA).

## Results

### Generation and production of nanobodies targeting mouse LYVE1

**Fig. 1:**
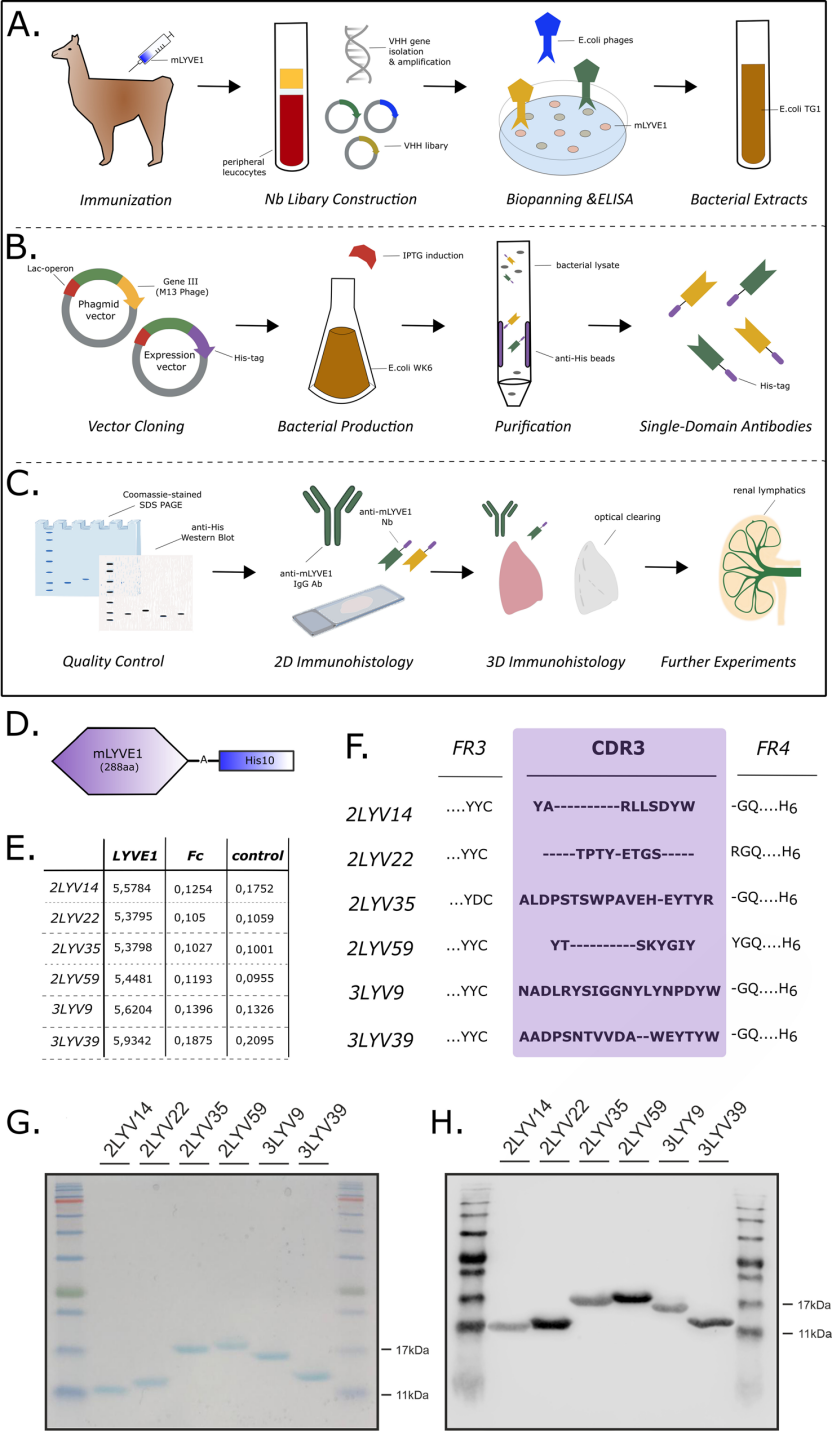
Generation and production of an anti-mouse LYVE1 nanobody. (**A**) To generate the anti-mouse LYVE1 nanobody, a recombinant mouse LYVE1 protein (288 amino acids) fused to a His10 tag (**D**) was injected into Ilama. Peripheral blood lymphocytes were harvested, and RNA extracted for cDNA synthesis. Nanobody-encoding sequences were amplified and cloned into phagemid vector pMECS, transformed into *E. coli* TG1 cells, resulting in a library of 109 independent transformants (*Library Construction*). LYVE1-specific nanobodies were enriched using phage display and biopanning on solid-phase coated mouse LYVE1. Binding performance was assessed by whole-cell phage ELISA (**E**). The wells were coated with mouse LYVE1 with His10 tag, mouse LYVE1 fused to human IgG1 Fc, human IgG1 Fc, or blocking buffer (control), allowing determination of clone specificity (*Biopanning & ELISA*). Bacteria carrying the anti-mouse LYVE1 nanobody plasmid were used for further steps (*Bacterial Extracts*). (**B**) Nanobody sequences were cloned into the pHEN6c expression vector with an additional 6x His tag (*Vector Cloning*). The amino acid sequences of the complementary determining regions (CDR) 3 and framework regions (FR) 3 and 4 of the six produced anti-mouse LYVE1 nanobodies are displayed (**F**). *E. coli* WK6 carrying pHEN6c-nanobody plasmids were cultured, and production was induced with IPTG (*Bacterial Production*). Bacterial cells were lysed, and nanobodies purified using His affinity chromatography (*Purification*). (**C**) Validation of LYVE1 nanobodies was performed using SDS-PAGE (**G**) and confirmed by anti-His western blotting (**H**), both of which detected proteins of the expected nanobody size (11–17 kDa). The anti-His western blotting specifically identified His-positive proteins. Quality control results confirmed successful production and purification (*Quality Control*). To assess the nanobodies’ suitability for immunolabelling, they were tested in two-dimensional (2D) histological sections and compared with anti-mouse LYVE1 IgG antibody (**Fig. ****S2**) (*2D Immunohistology*). They were further validated for wholemount immunolabelling and optical clearing (Fig. 2) (*3D Immunohistology*). Subsequently, nanobodies were used to investigate LYVE1 presence in various conditions (*Further Experiments*).

We selected LYVE1 as a target for nanobody generation, primarily as it is a candidate marker for lymphatic vessels across organs which, if labelled successfully, would enable 3D quantitative analysis of organ-specific lymphatic vascular networks in the body [40]. To generate nanobodies (Fig. 1A-C), peripheral blood lymphocytes were isolated from llamas that were repeatedly immunised with recombinant mouse LYVE1 protein fused with a polyhistidine (His10) tag (Fig. 1D). cDNA from all nanobody sequences present in peripheral blood lymphocytes, regardless of specificity for LYVE1, were amplified by PCR to generate a library of candidate nanobody clones. Nanobodies specific to mouse LYVE1 were enriched by biopanning. Based on ELISA data, six highly specific anti-LYVE1 nanobodies were chosen for production (Fig. 1E). The sequences of these six nanobodies (Fig. 1F) were cloned into an expression vector carrying a 6xHistidine tag and, after successful bacterial production, the nanobody clones were purified by His-affinity purification. We confirmed that successful purification was achieved with a single band observed between 11 and 17 kDa on Coomassie-blue stained SDS-PAGE gels (Fig. 1G) and following western blotting using anti-His Ab (Fig. 1H). A summary of our workflow is outlined in Fig. S1.

### Validation of anti-mouse LYVE1 nanobodies for 2D and 3D imaging

**Fig. 2:**
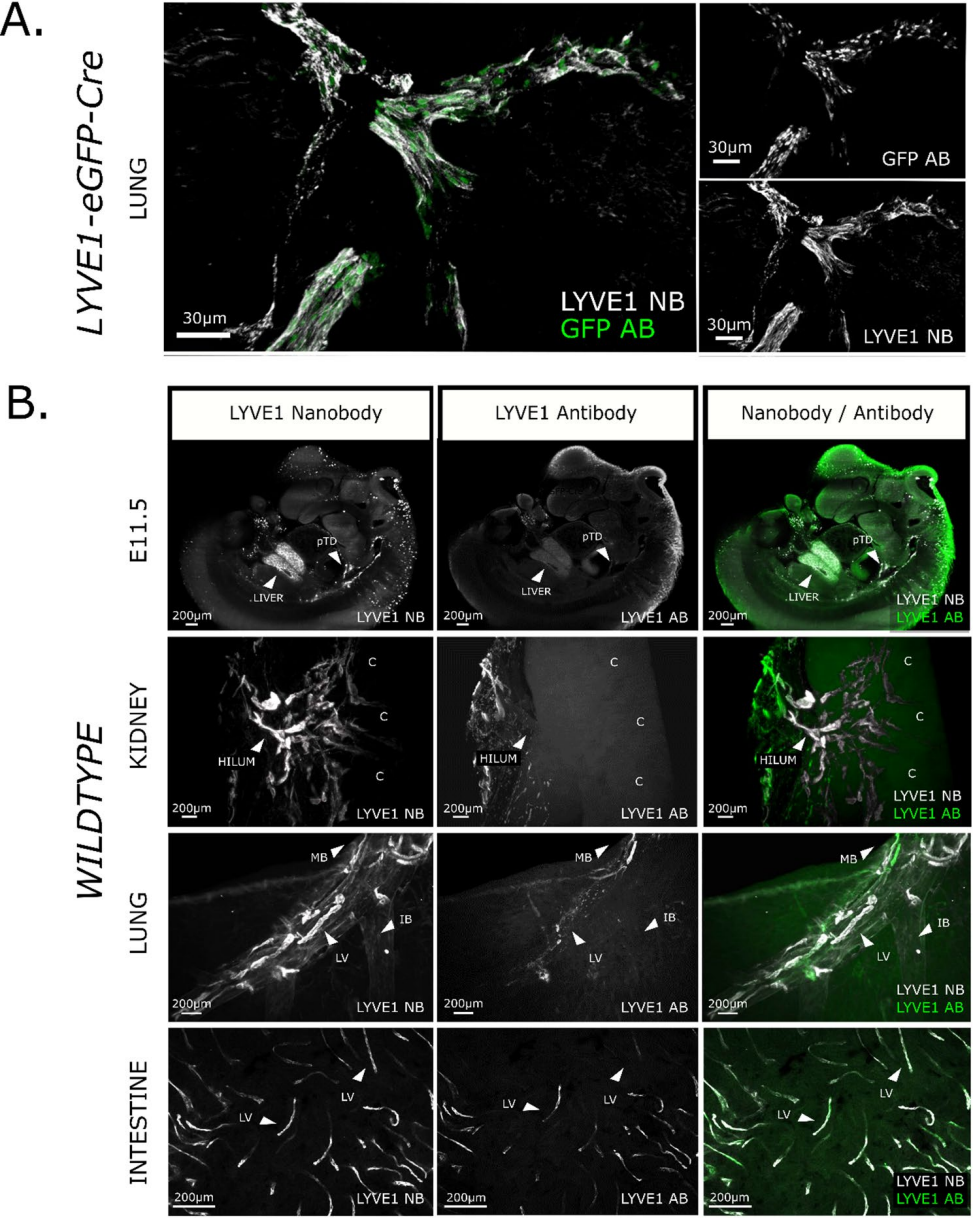
Characterisation and evaluation of an anti-mouse LYVE1 nanobody for 3D imaging of intact mouse organs. (**A**) Co-staining of anti-mouse LYVE1 nanobodies (white) and anti-GFP IgG antibodies (green) in P28 *Lyve1*^*eGFP − Cre*^ mouse lung tissue (*n* = 3) visualised by confocal microscopy show both markers highlighting a large lymphatic vessel. The anti-GFP IgG antibodies displays the expected nuclear staining pattern, while the LYVE1 nanobodies specifically label lymphatic endothelial cell surfaces. This reinforces the specificity of LYVE1 nanobodies demonstrated in 2D histology (**Fig. ****S2**) and supports their use in tissue-cleared specimens. Object-based colocalization *r* = 0.94 ± 0.04. Scale bar: 30 μm. Displayed as Imaris-based 3D renderings. (**B**) Efficacy of LYVE1 nanobodies in wholemount immunostaining is evaluated across various intact mouse organs using light sheet microscopy. In E11.5 mouse embryos (*n* = 6), LYVE1 nanobodies (white) distinctly visualise the primordial thoracic duct (pTD), whereas LYVE1 IgG antibodies (green) show a weak signal and a halo around the embryo surface, indicating limited penetration. Pearson correlation coefficient *r* = 0.73 ± 0.30. In P28 kidney (*n* = 6), nanobodies efficiently label lymphatic vessels beyond the superficial hilum, extending into the cortex (C), whereas IgG antibodies mainly highlight lymphatic vessels on the hilum surface (*r* = 0.47 ± 0.27). In P28 lung lobes (*n* = 6), nanobodies reveal more numerous and deeper lymphatic vessels (LV) with increased bronchial autofluorescence, in contrast to IgG antibodies, which primarily highlight vascular structures overlying the main bronchus (MB) (*r* = 0.50 ± 0.14). Additionally, nanobodies reveal vascular structures accompanying the intermediate bronchus (IB). In the small intestine (*n* = 6), confocal imaging shows more extensive staining of lymphatic vessels (LV) in the nanobody channel, while IgG antibodies detect fewer vessels, with lower intensity or only partially (*r* = 0.48 ± 0.13). Scale bar: 200 μm. Displayed as Imaris-based 3D renderings. E11.5 is displayed as a single z-stack slice.

Next, we evaluated the performance and specificity of the six anti-mouse LYVE1 nanobody clones. Initially, we performed conventional two-dimensional (2D) immunofluorescence, by labelling cryosections of C57BL/6 mice at E14.5 with either anti-LYVE1 nanobodies or a commercially available anti-LYVE1 IgG antibody and comparing patterns of expression. All six nanobody clones exhibited staining patterns overlapping with that of the LYVE1 antibody, successfully capturing primitive network of primordial thoracic ducts that give rise to systemic lymphatic vasculature [22, 46]. This was confirmed by Pearson correlation analysis with a mean ± SD of *r* = 0.94 ± 0.04.(Fig. S2A). To assess the specificity of anti-LYVE1 nanobodies in a 3D context, we performed nanobody labelling of optically-cleared lungs from reporter mice (*n* = 3) carrying Cre and an eGFP cassette within the endogenous *Lyve1* locus (*Lyve1*^*eGFP − Cre*^) [22, 35]. *Lyve1*^*eGFP − Cre*^ mice at P28 exhibited GFP expression in lymphatic endothelial cell nuclei, which overlapped with the cell surface labelling of our anti-LYVE1 nanobodies. Object-based colocalization analysis confirmed this quantitatively, with correlation coefficients of *r* = 0.94 ± 0.04 (Fig. 2A). These findings validate that the newly produced nanobodies recapitulate endogenous LYVE1 expression, demonstrating their specificity and suitability for 3D imaging of optically-cleared biological tissue.

### Detection of organ lymphatic vessels using anti-mouse LYVE1 nanobodies

Next, we tested whether using nanobodies targeting LYVE1 provides improvements in the detection of lymphatics in intact, optically cleared mouse organs, compared with conventional IgG antibodies. To do this, we performed wholemount immunolabelling of wildtype mouse embryos (*n* = 6) and a range of visceral organs at P28 (*n* = 6 per organ). We harvested intestine, a hollow viscous tissue amenable to wholemount techniques [47], alongside lung [48] and kidney [18] where lymphatics are located deep within the tissue, necessitating optical clearing prior to 3D imaging. Tissues were labelled using three anti-mouse LYVE1 nanobody clones (2LYV14, 2LYV22, 2LYV35) which exhibited the highest signal-to-background ratio in 2D staining experiments (Fig. S2B), or a commercially available anti-LYVE1 IgG antibody.

In all tissues examined, the commercial anti-LYVE1 IgG antibodies exhibited superficial staining, with weak immunoreactivity of lymphatic vessels deep within each tissue. E11.5 embryos additionally exhibited a halo of non-specific staining around the perimeter of the tissue. By contrast, the nanobodies penetrated deeper in both the embryos and organs, enhancing the detection of lymphatics in the E11.5 embryo and intact P28 intestine, lung, and kidney (Fig. 2B). Within E11.5 embryos, the nanobodies enabled visualisation of known LYVE1^+^ structures such as the primordial thoracic duct. Within the intact P28 kidney, nanobody labelling revealed interlobular lymphatic vessels extending from the hilum to the organ’s cortex [17, 18], whereas the conventional anti-LYVE1 IgG antibodies only labelled superficial hilar vessels (Video S1). Within the P28 lung parenchyma, lymphatic vessels running alongside the bronchi could be visualised in greater detail with nanobody labelling as compared with conventional antibodies (Video S2). Intestinal lymphatics also exhibited more widespread labelling with the nanobody approach (Fig. 2B).

To determine if non-lymphatic LYVE1^+^ structures were being captured, we further immunolabelled E18.5 kidneys with the myeloid marker, F4/80. LYVE1^+^ macrophages have been reported in embryonic kidneys [49] and indeed, using nanobody labelling, F4/80^+^ LYVE1^+^ cells were detected within E18.5 kidney (Fig. S3A). In these experiments, we also demonstrated a further methodological advantage over conventional IgG antibodies in that the incubation period with anti-LYVE1 nanobodies could be reduced to as little as four hours to achieve effective staining, whereas the equivalent staining with conventional anti-LYVE1 IgG antibodies was only visible after 48 h (Fig. S3B). When both nanobodies and IgG antibodies are incubated for four hours, only the nanobodies can visualise lymphatic vessels (Fig. S4A). Direct labelling of anti-LYVE1 nanobodies reduces the incubation period further, as staining is achieved in a single step (Fig. S4B). Overall, these findings show that anti-LYVE1 nanobodies penetrate mouse embryos and adult organs more rapidly and at a greater depth resulting in them being more efficacious than IgG antibodies for wholemount immunofluorescence, optical clearing and 3D imaging of lymphatics.

### Quantitative comparison and corroborative single-cell transcriptomic analysis reveal spatially and anatomically restricted expression of LYVE1 by mouse kidney lymphatics

Having successfully developed anti-LYVE1 nanobodies for wholemount immunofluorescence in mouse tissues, we hypothesised this novel reagent would enable us to examine LYVE1 expression and lymphatic architecture across a range of postnatal mouse organs. Therefore, we assessed heart, lung, skin and kidney at P28 in C57BL/6 wildtype mice (*n* = 6–8 mice per group), a timepoint where we reasoned that the developmental remodelling of lymphatics would be complete [4]. Focussing on imaging the parenchyma of each tissue, we undertook comparative 3D image analysis by utilising anti-LYVE1 nanobodies and an anti-podoplanin (PDPN) IgG antibody. PDPN is expected to label all postnatal lymphatic endothelial cells [50–52], serving as means of comparing the number of lymphatic vessels which also expressed LYVE1. Being a conventional IgG antibody, PDPN antibodies did not fully penetrate all organs, thus LYVE1 and PDPN vessel structures were extracted separately, binarized and analysed for geometric properties including vessel branch volume, length or mean radius using open-source 3D analysis software [39]. In regions of tissues fully penetrated by both immunolabels, PDPN should capture all lymphatic vessels, thus LYVE1^+^ vessels were examined relative to their PDPN^+^ counterparts (Fig. S5).

**Fig. 3:**
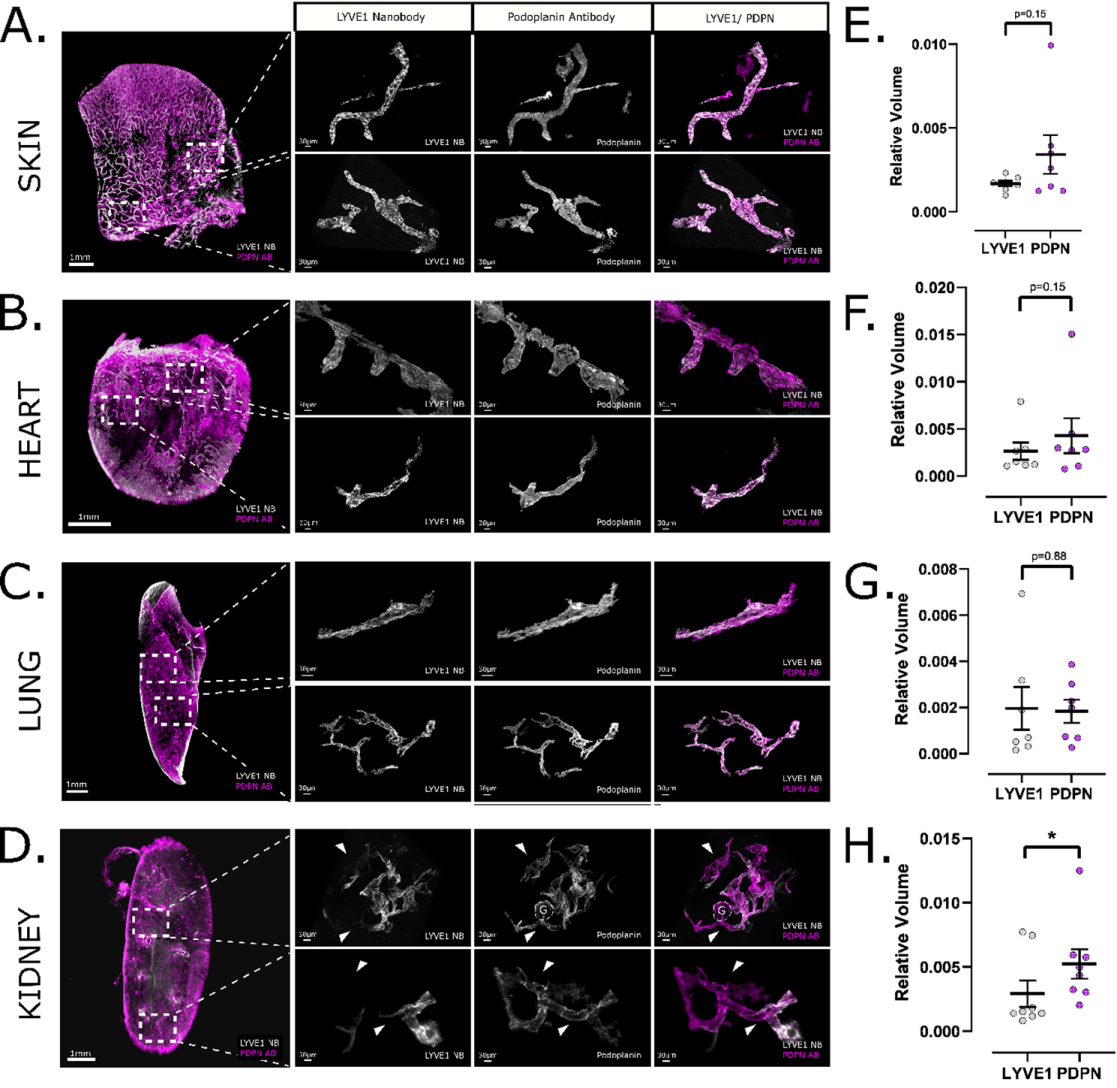
3D imaging of nanobody-stained mouse organs reveals organ-specific LYVE1 expression in kidney lymphatics. Co-staining of anti-mouse LYVE1 nanobodies (white) and anti-PDPN IgG antibodies (magenta) in C57BL/6 wild-type P28 organs by confocal microscopy. Each channel is displayed separately in grey scale. The scale bar in the overview is 1 mm and 30 μm for regions of interest. All images are presented as 3D reconstructions using Imaris software. (**A-D**) Comparison of the vascular structures visualised by anti-mouse LYVE1 nanobodies and anti-PDPN IgG antibodies in P28 skin, heart, and lung (**A-C**) reveal coherence between the lymphatic vessel markers LYVE1 and PDPN, as expected (Pearson correlation coefficient: heart *r* = 0.66 ± 0.08; lung *r* = 0.71 ± 0.11; skin *r* = 0.73 ± 0.06). In renal tissue (**D**) some vessel segments lack or have reduced levels of LYVE1 signal, indicated by arrows (*r* = 0.27 ± 0.05). PDPN, a marker for both lymphatic endothelial cells and podocytes, also highlights glomeruli (G), shown by dotted lines. (**E-H**) Quantitative analysis of LYVE1^+^ and PDPN^+^ lymphatic vessels in P28 organs. Each data point represents the average relative volume of 3–4 regions of interest imaged per animal. The error bar shows the standard error of means. An analysis of single regions of interest per data point can be found in **Fig. S7A-D**. Vessel volume has been adjusted to the overall sample volume. Paired student’s t-test found no significant difference between relative volumes of PDPN and LYVE1 in P28 skin (*n* = 6, *p* = 0.15, mean difference = 0.0017, t = 1.626, df = 6) (**E**), P28 heart (*n* = 7, *p* = 0.15, mean difference = 0.0016, t = 1.662, df = 6) (**F**) and P28 lung (*n* = 7, *p* = 0.88, mean difference = -0.00012, t = 0.1459, df = 6) (**G**). However, in P28 kidneys (*n* = 8), a significant difference was found (*p* = 0.039, mean difference = 0.002311, t = 2.528, df = 7) (**H**).

We inspected 3D images from P28 heart, lung, and skin, finding consistent overlap between PDPN and LYVE1 expression profiles in all organs examined (Fig. 3A-C). Accordingly, no significant difference between the relative volume of LYVE1^+^ vessels and PDPN^+^ vessels were found in the skin (*p* = 0.15), heart (*p* = 0.15) or lung (*p* = 0.88) (Fig. 3E-G). This was supported by Pearson correlation analysis, which showed comparable coefficients in skin (0.73 ± 0.06), heart (0.66 ± 0.08), and lung (0.71 ± 0.11), with no significant differences among these organs (*p* = 0,36). The Pearson correlation coefficients are most likely not closer to 1, as the two lymphatic markers are distributed differently along the vessel surface. This results in variations of voxel intensity, which affect the correlation analysis. However, the kidney showed a clear discrepancy. Analysing over 30 imaging volumes of individual vessel segments within the kidney’s parenchyma, we consistently identified kidney lymphatic vessels expressing PDPN, but lacking expression of LYVE1 (Fig. 3D). Quantitative analysis substantiated our findings, revealing a statistically significant difference (*p* = 0.03) between the relative volume of LYVE1^+^ vessels and that of PDPN^+^ vessels (Fig. 3H). In line with this, Pearson correlation coefficients were markedly lower in the kidney (0.27 ± 0.05), and significantly different from all other organs (*p* < 0.0001). These LYVE1^−^ PDPN^+^ vessels were always continuous with LYVE1^+^ PDPN^+^ lymphatics, demonstrating that they were part of the lymphatic network and not anatomically separate. The expression of LYVE1 in adjacent vessel branches suggests that the spatial restriction of LYVE1 expression by kidney lymphatics is not an artefact of limited immunolabel penetration.

The observation of LYVE1^−^ PDPN^+^ vessels within the kidney could represent anatomical hierarchy, as collecting vessels and lymphatic valve cells are known to lack LYVE1 expression [6]. To explore this further, we turned to a recent scRNA-seq dataset of 12-week-old wildtype mouse kidney [41]. We isolated 451 kidney lymphatic cells from this dataset and performed unsupervised sub-clustering, revealing four transcriptionally distinct sub-clusters (Fig. S6A). All sub-clusters expressed the canonical lymphatic markers, *Prox1*,* Vegfr3* and *Pdpn.* Contrastingly, only two of the four clusters expressed *Lyve1* (Fig. S6B). Examining differentially expressed genes (DEG) between these subclusters (Fig. S6C) to potentially identify marker genes (Fig. S6D), we identified one *Lyve1*^+^ subcluster enriched for lymphatic capillary markers *Ccl21a* (log_2_FC = 1.51, adjusted *p =* 3.14 × 10^− 25^) and *Reln* (log_2_FC = 2.37, adjusted *p =* 3.31 × 10^− 22^, whereas the second expressed *Aqp1* (log_2_FC = 3.87, adjusted *p =* 9.60 × 10^-41), *Ptx3* (log_2_FC = 4.74, adjusted *p =* 2.44 × 10^− 35^) and *Igfbp4* (log_2_FC = 1.48, adjusted *p =* 1.12 × 10^− 7^), akin to a recent murine study demonstrating a similar population cells localizing to lymphatic capillary terminals [53]. Conversely, of the two *Lyve1*^−^ subclusters, one was enriched for valve endothelial cell markers *Cldn11* (log_2_FC = 3.89, adjusted *p =* 1.02 × 10^− 30^) and *Foxc2* (log_2_FC = 2.82, adjusted *p =* 5.58 × 10^− 7^) [54, 55], whereas the fourth cluster expressed *Ackr4* (log_2_FC = 4.63, adjusted *p =* 1.26 × 10^− 24^) and *Foxp2* (log_2_FC = 1.18, adjusted *p =* 1.80 × 10^− 11^), associated with collecting vessel identity [56, 57]. Further examination of the transcriptome of this fourth subcluster using GO analysis (Fig. S6E) revealed terms related to immunomodulation including *negative regulation of dendritic cell differentiation* (GO:2001199, fold-enrichment = 86.5, FDR *=* 2.53 × 10^− 2^), *antigen processing and presentation of peptide antigen* (GO:0048002, fold-enrichment = 12.6, FDR *=* 9.44 × 10^− 3^) and *T cell differentiation* (GO:2001199, fold-enrichment = 6.1, FDR *=* 4.62 × 10^− 2^). A detailed list of differentially expressed genes between the different kidney lymphatic sub-clusters is provided as Table S2.

Collectively, a combination of nanobody labelling and 3D visualisation with quantitative analysis, corroborating by single-cell transcriptomic profiling, demonstrated spatially and anatomically restricted expression of LYVE1 by kidney lymphatics.

### Temporal decline in LYVE1 expression by kidney lymphatics occurs during postnatal maturation

**Fig. 4:**
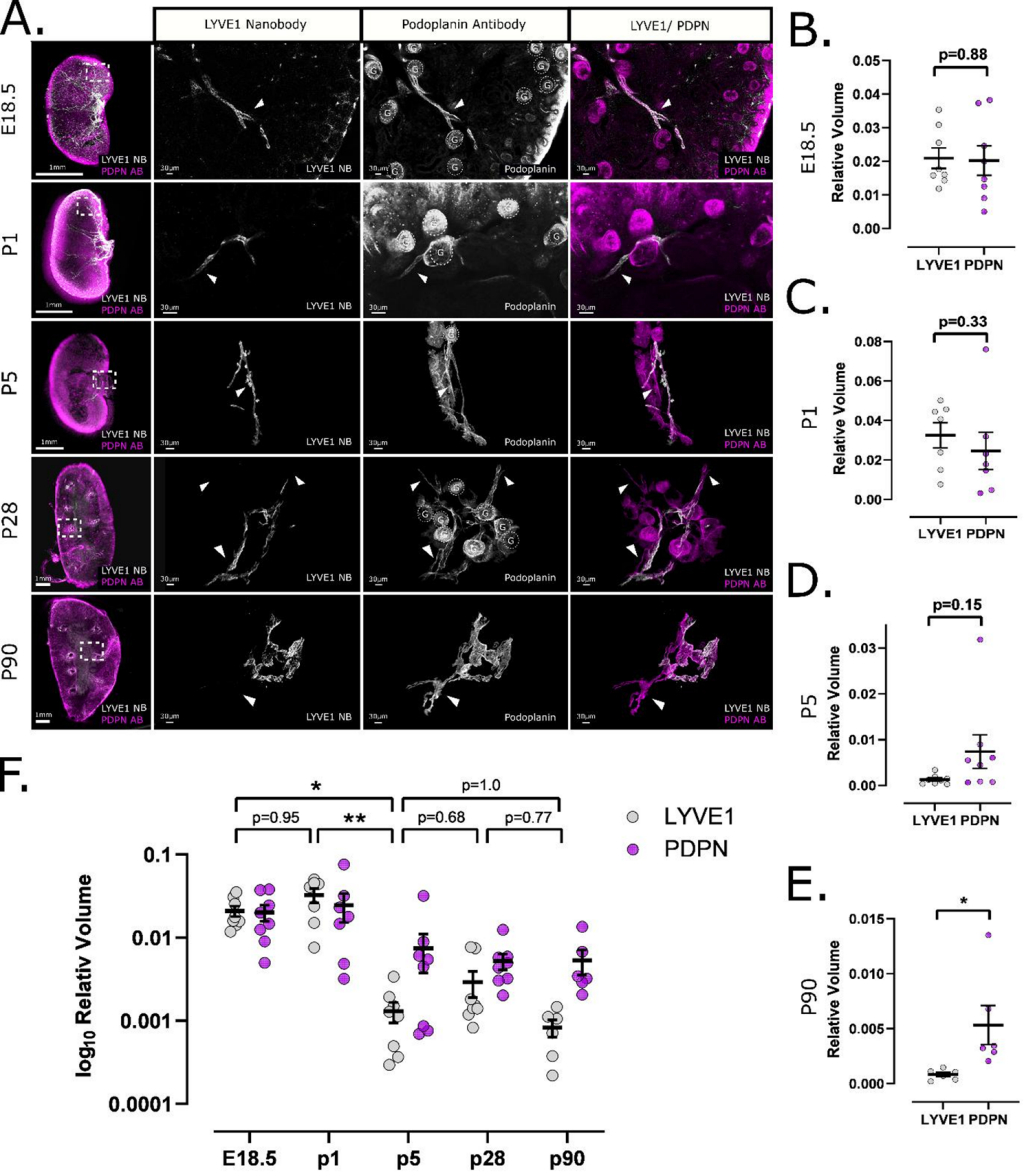
Spatial restricted expression of LYVE1 by kidney lymphatics occurs within the postnatal period. (**A**) Anti-mouse LYVE1 nanobodies (white) and anti-PDPN IgG antibodies (magenta) staining of C57BL/6 wild-type kidney tissue sections at various time points visualised by confocal microscopy. Each channel is displayed separately in grey scale. Regions of interest were visualised in areas of the kidney as indicated in the overview image. E18.5 and P1 regions of interest are presented as single z-stack slices. All other images are presented as 3D reconstructions using Imaris software. At E18.5 and P1, LYVE1 and PDPN equally visualise vascular structures with no detectable incoherence (Pearson correlation coefficient: E18.5 *r* = 0.85 ± 0.04; P1 *r* = 0.79 ± 0.05). By P5, differences in staining patterns between LYVE1 and PDPN appear, though fewer vessels and regions of interest show incoherence compared to P28 (*r* = 0.33 ± 0.13) (Fig. 3D). P90 kidney lymphatics exhibit a similar number of LYVE1^−^ areas, particularly in vessels of decreasing size (*r* = 0.30 ± 0.07). Arrows indicate LYVE1^−^ vessel segments. Glomeruli (G) visualised by the PDPN IgG antibody are outlined by dotted lines. Scale bars: 1 mm (overview), 30 μm (regions of interest). (**B-F**) Quantitative analysis of LYVE1^+^ and PDPN^+^ lymphatic vessels in kidneys. Each datapoint represents the average relative volume of 3–4 regions of interest imaged per kidney, except for E18.5 and P1 which were imaged as a complete unit. Error bar shows standard error of means. Vessel volume was adjusted to overall sample volume. Single region analyses are available in Fig. S7E-F. Paired student’s t-test showed no significant difference between LYVE1 and PDPN in E18.5 (*p* = 0.88) (**B**), P1 (*p* = 0.33) (**C**) and P5 (*p* = 0.15) (**D**). However, in P90 kidneys, a significant difference was found (*p* = 0.049) (**E**). (**F**) Statistical analysis of LYVE1 volume dynamics throughout kidney maturation was conducted using nonparametric multiple comparisons for relative contrast effect testing [45] across timepoints, revealing a significant overall alteration (*p* = 0.0004). No significant differences were found between E18.5 and P1 (*p* = 0.95), P5 and P28 (*p* = 0.68), P5 and P90 (*p* = 1.0), or P28 and P90 (*p* = 0.77). However, a significant decline was observed between P1 and P5 (*p* = 0.002). Statistical analysis of single regions of interest is provided in Fig. S7G

Finally, given the characteristic nature of restricted LYVE1 expression by lymphatics in the kidney, we sought to gain insight into the dynamics of LYVE1 expression over the course of kidney development and maturation. We characterised and compared 3D imaging of wildtype kidney lymphatics at five timepoints: E18.5, P1, P5, P28 and P90. E18.5 (*n* = 8) was chosen as an embryonic timepoint where we have previously characterised the spatial relationships of kidney lymphatics, and during which LYVE1 is expressed by all bona fide lymphatic vessels [40]. Between P1 (*n* = 7) and P5 (*n* = 8), the development of nephrons; the functional units of the kidney, has been reported to reach cessation [58] with P28 (*n* = 8) representing early adulthood and P90 (*n* = 6) representing a mature stage by which time mouse kidneys have reached their full size. At E18.5, all PDPN^+^ lymphatic vessels were found to express LYVE1 and, accordingly, quantitative analysis demonstrated no significant differences between LYVE1^+^ vessel and PDPN^+^ vessel volume (*p* = 0.88, mean difference = -0.0007419, t = 0.1533, df = 7) (Fig. 4A-B). Likewise, there was no significant difference in LYVE1^+^ vessel and PDPN^+^ vessel volume at P1 (*p* = 0.33, mean difference = -0.007961, t = 1.046, df = 6) (Fig. 4A, C), or P5 (*p* = 0.15, mean difference = 0.006101, t = 1.642, df = 7), albeit there were some visible regions containing LYVE1^−^ PDPN^+^ lymphatic vessels at this early postnatal timepoint (Fig. 4A, D). Thereafter, there was a striking decrease in LYVE1^+^ regions of PDPN^+^ lymphatic vessels as the mice aged (Fig. 4A). By P28, there was a significant difference between LYVE1^+^ vessel and PDPN^+^ vessel volume (*p* = 0.03, mean difference = 0.002311, t = 2.528, df = 7) (Fig. 3H), consistent with findings at P90 (*p* = 0.04, mean difference = 0.004502, t = 2.574, df = 5) (Fig. 4E). Non-parametric multiple comparisons (estimation method = global pseudo ranks, type of contrast = Tukey, confidence level = 95%) were applied to evaluate LYVE1^−^ vessels across the five different timepoints. Using this approach, an overall significant alteration could be revealed (*p* = 0.0004, quantile = 2.74). No significant differences could be found between time points E18.5 and P1 (*p* = 0.95), nor between P28 and P90 (*p* = 0.77) or P5 and P28 (*p* = 0.68) (Fig. 4F). However, in an analysis of individual regions of interests across all animals, a significant difference between P5 and P28 was detected (*p* = 0.03) (Fig. S7G). The most pronounced difference in this analysis within maturation stages occurred between P1 and P5 (*p* = 0.002) (Fig. 4F). Further, significant differences were also noted between E18.5 and P28 (*p* = 0.0004), P1 and P28 (*p* = 0.003), and P1 and P90 (*p* = 0.0013). This was supported by Pearson correlation analysis, which showed high correlation between LYVE1 and PDPN at early stages (E18.5: 0.85 ± 0.04; P1: 0.79 ± 0.05), followed by a marked decrease at later stages (P5: 0.33 ± 0.13; P28: 0.28 ± 0.09; P90: 0.30 ± 0.07). One-way ANOVA confirmed a significant overall decline in correlation across developmental timepoints (*p* < 0.0001), with post-hoc Tukey comparisons identifying the most pronounced decrease between P1 and P5 (*p* < 0.0001). In summary, our 3D quantitative analysis of LYVE1 expression during kidney lymphatic vessel remodelling revealed that the organ-specific feature of restricted LYVE1 expression manifests during the organ’s early postnatal maturation, occurring most rapidly between P1-P5.

## Discussion

In this study, we successfully generated and validated novel anti-LYVE1 nanobodies to improve 3D imaging of lymphatic vessels in mice. Our approach enabled faster and more effective wholemount immunostaining of intact murine organs, facilitating both qualitative and quantitative analysis of organ-specific lymphatic architecture. Testament to the potential of this novel tool for discovery, we found that LYVE1 expression was spatially restricted in lymphatic vessels of the kidney as compared to other organs, and that this phenomenon manifests during the organ’s postnatal maturation, coinciding with the cessation of organogenesis in the kidney. In developing a reagent for 3D lymphatic imaging, we have therefore highlighted a characteristic feature of lymphatics in the mouse kidney.

Previous reports have harnessed the beneficial properties of single-domain antibodies for 3D imaging, specifically their small size and improved epitope detection compared with conventional IgG antibodies [59], high solubility, heat stability, chemical resistance [30] and cost-effectiveness [60]. Recent examples of 3D imaging utilising nanobodies include labelling human dermal vasculature [36], capturing GFP^+^ cells in whole mice [27] or characterising the murine placental vasculature [61]. Such nanobodies surmount the challenges of using conventional IgG antibodies for immunolabelling, including long incubation times or specialised equipment being required for tissue penetration [28]. To our knowledge, this represents the first nanobody that binds to lymphatics. LYVE1 was selected given its reported widespread expression on lymphatic vessels [1], and its amenability to successful nanobody production given its simple structure and limited number of post-translational modifications compared to other lymphatic markers such as PDPN. The nanobody clones presented in this report have other applications beyond 3D imaging, including flow cytometry and cell isolation, live imaging or functional blocking experiments in mice [62–67]. The versality and rapid staining achievable by nanobodies also complements the recent advent in optical clearing of live biological tissues [68–70], enabling visualisation of LYVE1-expressing cells in real-time.

Having generated and validated anti-LYVE1 nanobodies, we demonstrate whole organ imaging of lymphatic vascular networks. Although previous reports have utilised LYVE1 as a marker in isolation to capture lymphatic vessels [49, 71], nanobody labelling revealed that LYVE1 expression is spatially restricted by lymphatics of the kidney from an early postnatal stage. Given the location of these LYVE1^−^ PDPN^+^ vessels and their scarcity in other organs, we provide evidence for a characteristic feature of lymphatics in the mouse kidney. We have recently shown that these findings also translate to human tissues, as 3D imaging of human kidneys demonstrates regions of lymphatic vessels with LYVE1^−^ PDPN^+^ expression [25] as well as scRNA-seq revealing that approximately 30% of kidney lymphatic cells lack *LYVE1* expression [25]. As suggested by the mouse kidney scRNA-seq data in this paper, these *Lyve1*^−^ vessels, may have a collecting vessel identity. However, given the absence of smooth muscle lining LYVE1^−^ lymphatics in the human kidney, and parallel findings of LYVE1^−^ lymphatic capillaries in the nasal mucosa [72], these vessels could alternatively represent an atypical capillary phenotype, adding to the complexity of organ-specific molecular heterogeneity of lymphatics. This demonstration of inter-organ and intra-organ molecular heterogeneity of lymphatics may also apply to other contexts [6]. The anti-mouse LYVE1 nanobodies used in this study thus provide a powerful tool to uncover previously undetectable aspects of lymphatic biology and pathophysiology in the kidney. These findings also demonstrate the importance of exercising caution when using single markers to discriminate lymphatics. Utilisation of LYVE1 in isolation misses a proportion of lymphatic vessels in the kidney. Moreover, in pathological conditions, lymphatic endothelium may shed LYVE1 expression [73–75], further necessitating the use of multiple markers to discriminate lymphatics, particularly in contexts where the anatomical distribution or phenotype of lymphatics within an organ is not well understood.

Our study is not without limitations. Firstly, our validation of nanobodies in 3D imaging data is performed with co-labelling of conventional IgG antibodies. As a result, experiments are limited by the penetration depth of full-size IgG antibodies, which necessitated the use of tissue sections (0.5–2 mm thick) instead of entire organs and separate analysis of PDPN^+^ and LYVE1^+^ vessels for quantitative evaluation. Secondly, the nanobodies generated bind to mouse LYVE1, but to our knowledge, do not exhibit reactivity with human LYVE1. Moreover, our study does not address the functional significance of lymphatic heterogeneity, which is out of the scope of this technical report. CD44^+^ immune cells enter lymphatics *via* hyaluronan-mediated binding to LYVE1 on lymphatic endothelium [32, 76], thus the limited expression of LYVE1 on kidney lymphatics may have implications for renal immune cell trafficking [77, 78]. Akin to the findings presented in our paper, single-cell transcriptomics of the mouse nasal mucosa indicates that LYVE1^−^ lymphatic cells are enriched for expression of molecules involved in immunomodulation [72], suggesting that this subpopulation may be directly involved in local immunity. Finally, deciphering the origins of lymphatic heterogeneity remains a subject of ongoing debate. Our findings support a developmental origin of restricted LYVE1 expression, as we observe the greatest increase in the proportion of LYVE1^−^ vessel volume during a postnatal window during which kidney organogenesis ceases. Thus, differences in organ-specific lymphatic development, including paracrine or physical signals from tissue progenitors, transcriptional enhancers or repressors and alternative cellular lineages are all potential contributors to structural, molecular and functional heterogeneity of lymphatic vessels [79].

## Conclusion

In conclusion, we report the generation of the first nanobodies targeting lymphatic vessels. We find that anti-LYVE1 nanobodies represent a promising and simple-to-use tool to structurally profile organ lymphatics in mouse, rendering the complex technique of 3D imaging more widely accessible within the rapidly evolving field of lymphatic biology. Testament to the utility of these nanobodies, we add to the evidence that lymphatic vessels within certain organs possess unique anatomical and molecular properties, with spatially restricted expression of LYVE1 upon lymphatics in the mouse kidney. Overall, we anticipate nanobodies will contribute to the suite of novel experimental tools advancing the understanding of lymphatic heterogeneity in health and disease.

## Supplementary Information

Below is the link to the electronic supplementary material.

Supplementary Material 1

Supplementary Material 2

Supplementary Material 3

Supplementary Material 4

## Data Availability

The script for scRNA-seq analysis is publicly available at DJ’s Github account ( https://github.com/daniyal-jafree1995 ). The scRNA-seq dataset, derived from a previous study (41), is available upon request to the study’s corresponding authors and will be made publicly available upon publication of the original study. The data that support the findings of this study are available from the corresponding author upon reasonable request. The anti-mouse LYVE1 nanobodies developed in this study will be made commercially available through a collaboration with LIMAA Technologies GmbH, Berlin, Germany ( https://www.nanobodies-online.com/ ).
