# Supplementary material for: Nanobody Immunolabelling and three-dimensional imaging reveals spatially restricted LYVE1 expression by kidney lymphatic vessels in mice
Source: J Nanobiotechnology. 2025 Oct 13;23:667. doi: 10.1186/s12951-025-03759-3 (PMC12519744; doi:10.1186/s12951-025-03759-3)
Supplement: Supplementary file 4 — Supplementary Material 4 [file 12951_2025_3759_MOESM4_ESM.pdf]

Supplementary Materials:

Nanobody immunolabelling and three-dimensional imaging reveals spatially restricted LYVE1 expression by kidney lymphatic vessels in mice

Eva Maria Funk[1,2,3], Daniyal J Jafree[4,5,6], Nils Rouven Hansmeier[1,2,7], Clàudia Abad Baucells[1,2,8], Rose Yinghan Behncke[1,2], Gideon Pomeranz[4,5], Maria Kolatsi-Joannou[4,5], William J Mason[4,5], Dale Moulding[4], Lauren G Russell[4,5], Ayshwarya Subramanian[9], Sascha Ulferts[1,2], Laura Wilson[4,5], David A Long[4,5*], René Hägerling[1,2,6,10*†]

[1] Research Group 'Lymphovascular Medicine and Translational 3D-Histopathology', Institute of Medical and Human Genetics, Charité-Universitätsmedizin Berlin, Berlin, Germany; [2] Berlin Institute of Health at Charité-Universitätsmedizin Berlin, BIH Center for Regenerative Therapies, Berlin, Germany; [3] Berlin Institute of Health at Charité –Universitätsmedizin Berlin, BIH Biomedical Innovation Academy, BIH-MD Stipendium Program, Berlin, Germany; [4] Developmental Biology & Cancer Research & Teaching Department, UCL Great Ormond Street Institute of Child Health, UCL, London, UK; [5] UCL Centre for Kidney and Bladder Health, University College London, London, UK; [6] Wellcome Trust Sanger Institute, Hinxton, UK; [7] Research Group Development and Disease, Max Planck Institute for Molecular Genetics, Berlin, Germany; [8] Department of Nephrology and Medical Intensive Care, Charité Universitätsmedizin Berlin, Berlin, Germany; [9] Department of Molecular Biology and Genetics, College of Arts and Sciences, Cornell University, Ithaca, NY, USA; [10] Berlin Institute of Health at Charité – Universitätsmedizin Berlin, BIH Academy, Clinician Scientist Program, Berlin, Germany.

* These authors contributed equally as senior authors.

† Author to whom correspondence should be addressed (rene.haegerling@charite.de).

Figure	nanobody	1. IgG antibody	2. IgG antibody
**Fig.2A**	Anti-mouse LYVE1 Nb Mix [5µg/ml] [4 h]	rabbit monoclonal anti-His antibody [1:200] [5 days]	anti-rabbit IgG Alexa Fluor™ 647 [1:1000] [overnight]
		chicken polyclonal anti-GFP [1:200] [5 days]	anti-chicken highly cross-absorbed Alexa Fluor™ 488+ [1:1000] [overnight]
**Fig. 2B**	Anti-mouse LYVE1 Nb Mix [1µg/ml] [10 days]	rabbit monoclonal anti-His antibody [1:200] [10 days]	anti-rabbit IgG Alexa Fluor™ 647 [1:1000] [9 days]
		goat polyclonal anti-mouse LYVE1 [1:100] [10 days]	anti-goat IgG Highly cross-absorbed Alexa Fluor™ 488+ antibody [1:1000] [9 days]
**Fig. 3**	Anti-mouse LYVE1 Nb Mix [5µg/ml] [1 day]	rabbit monoclonal anti-His antibody [1:200] [5 days]	anti-rabbit IgG Alexa Fluor™ 647 [1:1000] [2 days]
		hamster monoclonal anti-Podoplanin [1:200] [5 days]	anti-Syrian hamster IgG Cross-Absorbed Alexa Fluor™ 546 [1:1000] [2 days]
**Fig. 4**	Anti-mouse LYVE1 Nb Mix [5µg/ml] [1 day]	rabbit monoclonal anti-His antibody [1:200] [5 days]	anti-rabbit IgG Alexa Fluor™ 647 [1:1000] [2 days]
		hamster monoclonal anti-Podoplanin [1:200] [5 days]	anti-Syrian hamster IgG Cross-Absorbed Alexa Fluor™ 546 [1:1000] [2 days]
**Fig. 4 (E18.5 + P1)**	Anti-mouse LYVE1 Nb Mix [5µg/ml] [overnight]	rabbit monoclonal anti-His antibody [1:200] [2 days]	anti-rabbit IgG Alexa Fluor™ 647 [1:1000] [overnight]
		hamster monoclonal anti-Podoplanin [1:200] [2 days]	anti-Syrian hamster IgG Cross-Absorbed Alexa Fluor™ 546 [1:1000] [overnight]
**Fig. S2A**	Anti-mouse LYVE1 Nb single clones [0.1µg/ml] [overnight]	rabbit monoclonal anti-His antibody [1:200] [overnight]	anti-rabbit IgG Alexa Fluor™ 647 [1:1000] [1h]
		goat polyclonal anti-mouse LYVE1 [1:100] [overnight]	anti-goat IgG Alexa Fluor™ 568 [1:1000] [1h]
**Fig. S3A**	Anti-mouse LYVE1 Nb Mix [5µg/ml] [4h]	rabbit monoclonal anti-His antibody [1:200] [10 days]	anti-rabbit IgG Alexa Fluor™ 647 [1:1000] [overnight]
		rat monoclonal anti-F4/80 antibody [1:50] [10 days]	anti-rat Highly cross-absorbed Alexa Fluor™ 488+ antibody [1:1000] [overnight]

**Fig. S3B**	Anti-mouse LYVE1 Nb single clones [0.1µg/ml] [4h]	rabbit monoclonal anti-His antibody [1:200] [2 days]	anti-rabbit IgG Alexa Fluor™ 647 [1:1000] [overnight]
		goat polyclonal anti-mouse LYVE1 [1:100] [2 days]	anti-goat IgG Highly cross-absorbed Alexa Fluor™ 488+ antibody [1:1000] [overnight]
**Fig. S4A**	Anti-mouse LYVE1 Nb Mix [5µg/ml] [4h]	Alexa Fluor™ Zenon labelled rabbit monoclonal anti-His antibody [1:200] [1 day]	
		goat polyclonal anti-mouse LYVE1 [1:100] [4h]	anti-rabbit IgG Alexa Fluor™ 647 [1:1000] [1 day]
**Fig. S4B**	Alexa Fluor™ 647 NHS Ester labelled anti-mouse LYVE1 Nb Mix [1µg/ml] [1 day]		
		goat polyclonal anti-mouse LYVE1 [1:100] [7 days]	anti-goat IgG Highly cross-absorbed Alexa Fluor™ 488+ antibody [1:1000] [5 days]

Supplementary Table 1: Overview of antibodies, incubation times, and concentrations for immunostaining experiments

Antibodies used for each staining experiment are listed together with concentration/dilution and incubation time. Experiments are indicated by figures, which present the according results.

Supplementary Table 2. Comprehensive list of differentially expressed genes between kidney lymphatic sub-clusters identified by single-cell RNA sequencing

Detailed overview of all differentially expressed genes between different kidney lymphatic sub-clusters described in the single-cell RNA sequencing dataset. Due to the size of the table, it is provided as a separate file.

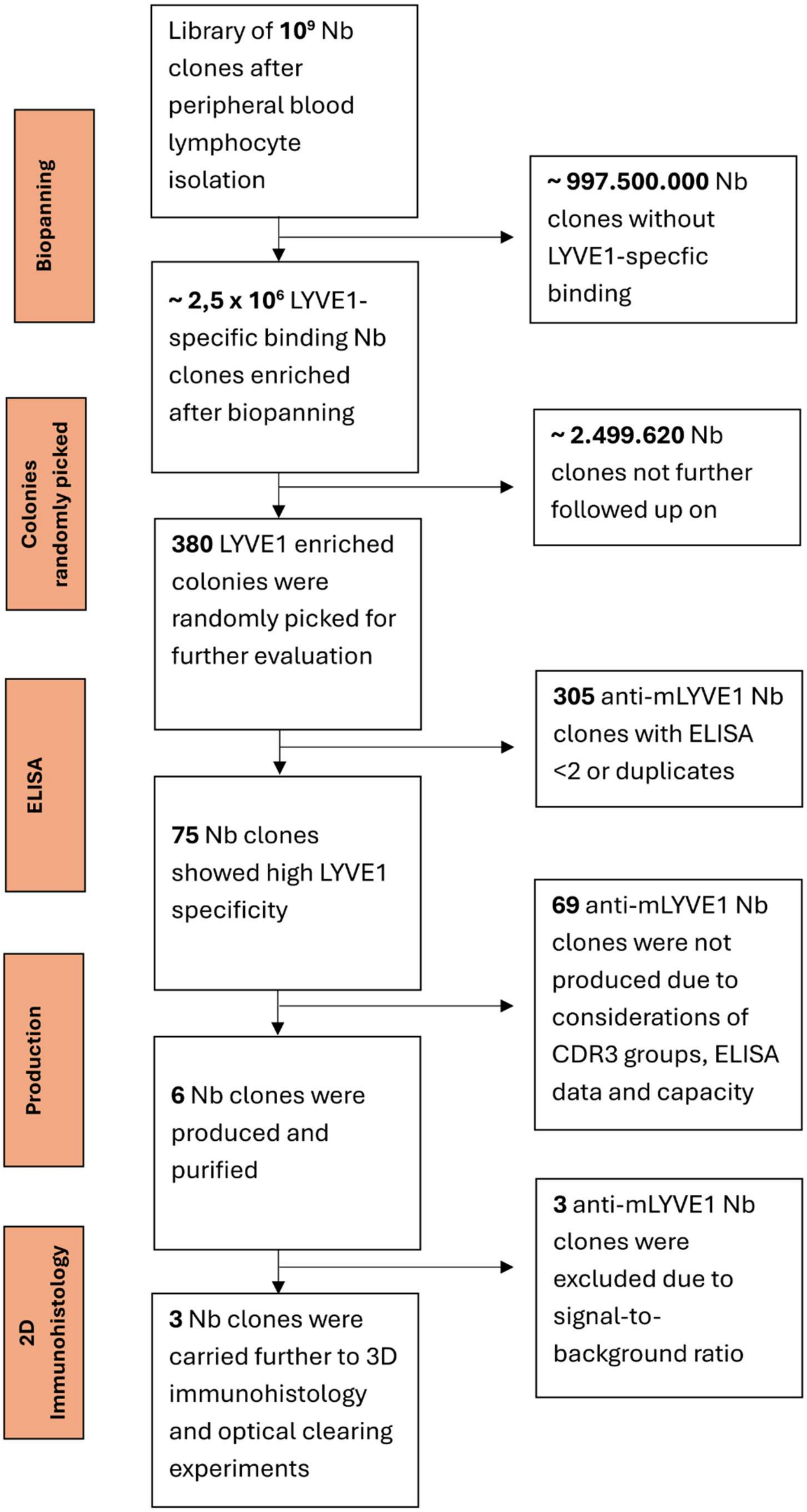
Biopanning
Colonies randomly picked
ELISA
Production
2D Immunohistology
Library of 10^9 Nb clones after peripheral blood lymphocyte isolation
~ 997.500.000 Nb clones without LYVE1-specfic binding
~ 2,5 x 10^6 LYVE1-specific binding Nb clones enriched after biopanning
~ 2.499.620 Nb clones not further followed up on
380 LYVE1 enriched colonies were randomly picked for further evaluation
305 anti-mLYVE1 Nb clones with ELISA <2 or duplicates
75 Nb clones showed high LYVE1 specificity
69 anti-mLYVE1 Nb clones were not produced due to considerations of CDR3 groups, ELISA data and capacity
6 Nb clones were produced and purified
3 anti-mLYVE1 Nb clones were excluded due to signal-to-background ratio
3 Nb clones were carried further to 3D immunohistology and optical clearing experiments

Supplementary Fig. 1 Flowchart of anti-mouse LYVE1 nanobody clone selection process

After initial immunization and establishment of a nanobody library based on peripheral blood leucocytes, a total of 10^9 potential nanobody clones were available. After 400-fold enrichment by phage display and biopanning of LYVE1-spefic binding clones, 380 clones were randomly selected for ELISA analysis. 305 clones scored <2, which was set as the cut-off for promising mouse LYVE1 specificity or were duplicates. From the remaining 75 highly LYVE1 specific nanobody clones, due to capacity reasons, six clones were selected based on ELISA data and diversity of CDR3 groups for production. All six clones successfully immunolabelled LYVE1 in 2D tissue sections, but due to differences in signal-to-background ratio, three clones were finally selected for 3D immunostaining with optical clearing.

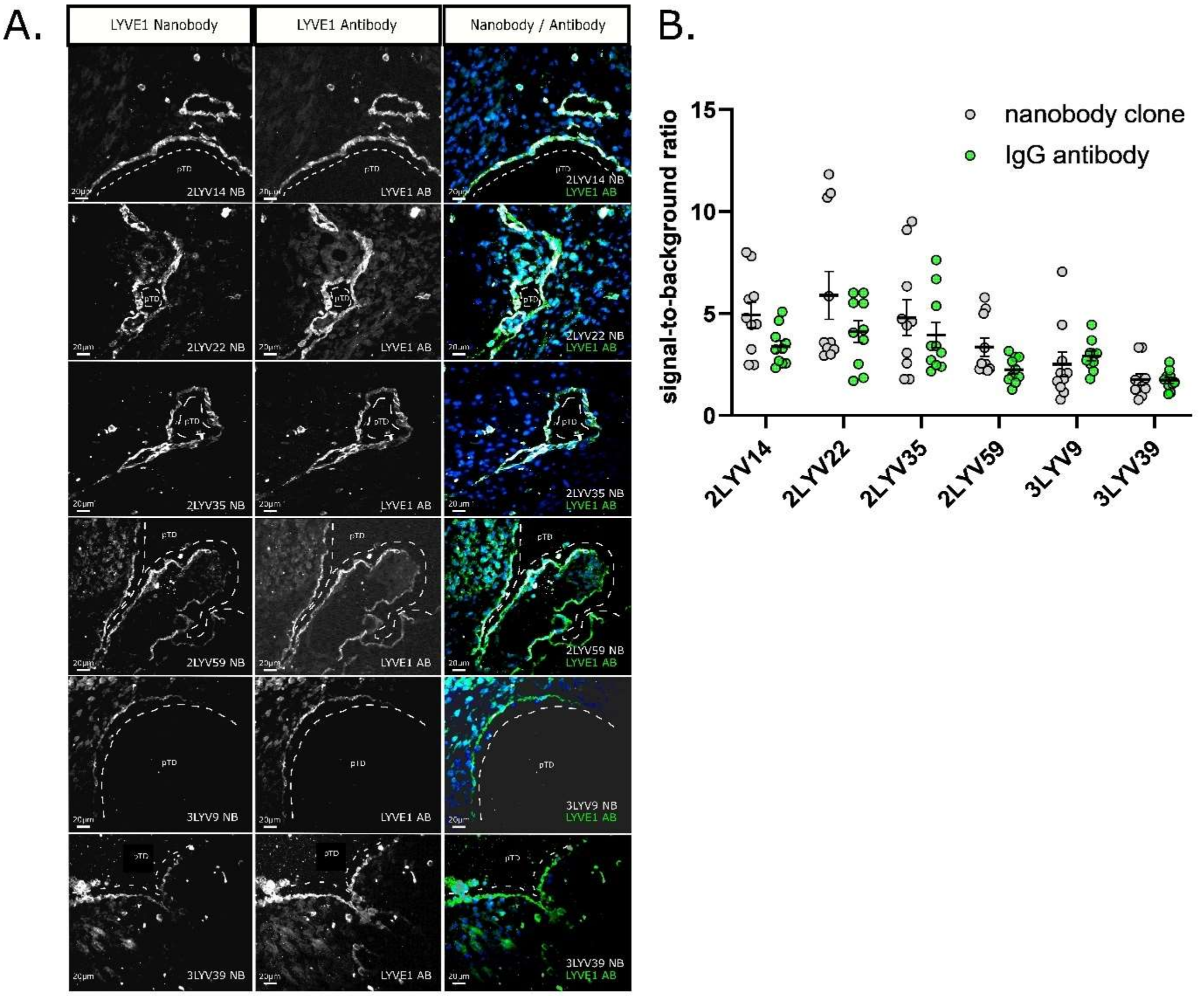

Supplementary Fig. 2 Validation of anti-mouse LYVE1 nanobody for 2D immunolabeling

(**A**) LYVE1 staining of 5 µm cryosections of E14.5 mouse embryos was performed using anti-mouse LYVE1 nanobody clones (white) and IgG antibodies (green), with separate channels displayed in grayscale. The nanobody clones are indicated in each image corner. Hoechst 33342 (blue) was used to verify the staining process and is visible in the merged image. Dashed lines outline the primordial thoracic duct (pTD). Single cells not adjacent to the lymphatic vasculature were detected alongside vascular structures. All six nanobody clones visualised lymphatic structures comparably to IgG controls. Pearson correlation coefficients: r = 0.94 (2LYV14), r = 0.97 (2LYV22), r = 0.92 (2LYV35), r = 0.87 (2LYV59), r = 0.98 (3LYV9), r = 0.98 (3LYV29). Scale bar 20 µm. (**B**) A quantitative comparison of the signal-to-background ratios for the six anti-mouse LYVE1 nanobody clones in 2D immunofluorescence was performed by selecting ten random values from signal and background areas in ImageJ. Mean values and SEM for nanobody clones and their corresponding IgG antibodies are as follows: for 2LYV14, nanobody clone 4.93 ± 0.62 and IgG antibody 3.38 ± 0.93; for 2LYV22, nanobody clone 5.90 ± 1.18 and IgG antibody 4.12 ± 1.66; for 2LYV35, nanobody clone 4.80 ± 0.88 and IgG antibody 3.94 ± 1.93; for 2LYV59, nanobody clone 3.35 ± 0.45 and IgG antibody 2.24 ± 0.63; for 3LYV9, nanobody clone 2.52 ± 0.60 and IgG antibody 2.92 ± 0.75; and for 3LYV39, nanobody clone 1.76 ± 0.28 and IgG antibody 1.74 ± 0.48. Statistical comparisons between single nanobody clones and corresponding IgG antibodies (multiple t-tests, Bonferroni-Dunn correction) showed no significant differences ($p > 0.05$).

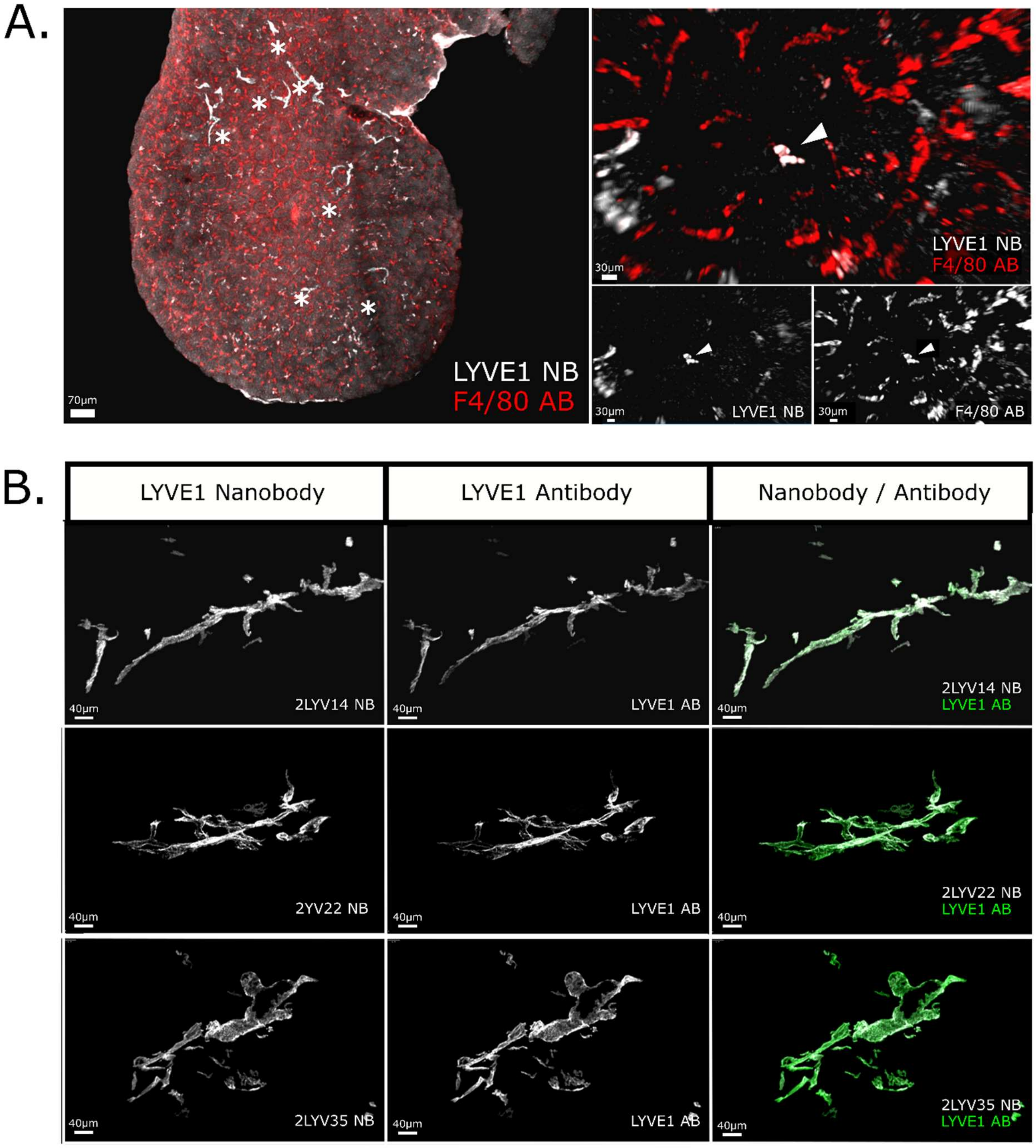

Supplementary Fig. 3 Validation of anti-mouse LYVE1 nanobody in optically-cleared murine kidney after incubation period reduction and validation of myeloid cell expression

(**A**) Co-staining of anti-mouse LYVE1 nanobodies (white) and anti-F4/80 IgG antibodies (red) in C57BL/6 wildtype E18.5 kidney, visualised by confocal microscopy. The macrophage marker F4/80 successfully detected multiple cells within the kidney, while next to lymphatic vessels (*), the anti-mouse LYVE1 nanobodies labelled occasional single cells not connected to the

lymphatic vasculature. These cells are co-labelled by anti-F4/80 IgG antibodies, suggesting that the nanobodies are not only capable of labelling $LYVE1^+$ lymphatic vessels but can further be used to study $LYVE1^+$ macrophage subsets. Pearson correlation coefficient r= 0,37. The scale bar is 70 µm, 30 µm respectively. Images are presented as 3D reconstructions using Imaris software.

(**B**) Anti-mouse LYVE1 nanobody clones 2LYVE14, 2LYVE22, and 2LYVE35 used in immunostaining of 0.5–2 mm thick P28 murine kidney sections visualised by confocal microscopy. Control anti-mLYVE1 IgG antibodies were incubated for 48 h, in comparison to an incubation period of 4 h for nanobodies. The nanobodies (white) successfully detected the identical biological structures as the anti-mouse LYVE1 IgG antibody control staining (green), despite a noticeable reduction of incubation time by 44h. Pearson correlation coefficients: r = 0.84 (2LYVE14), r = 0.77 (2LYVE22), r = 0.86 (2LYVE35).The scale bar is 40 µm. Images are presented as 3D reconstructions using Imaris software.

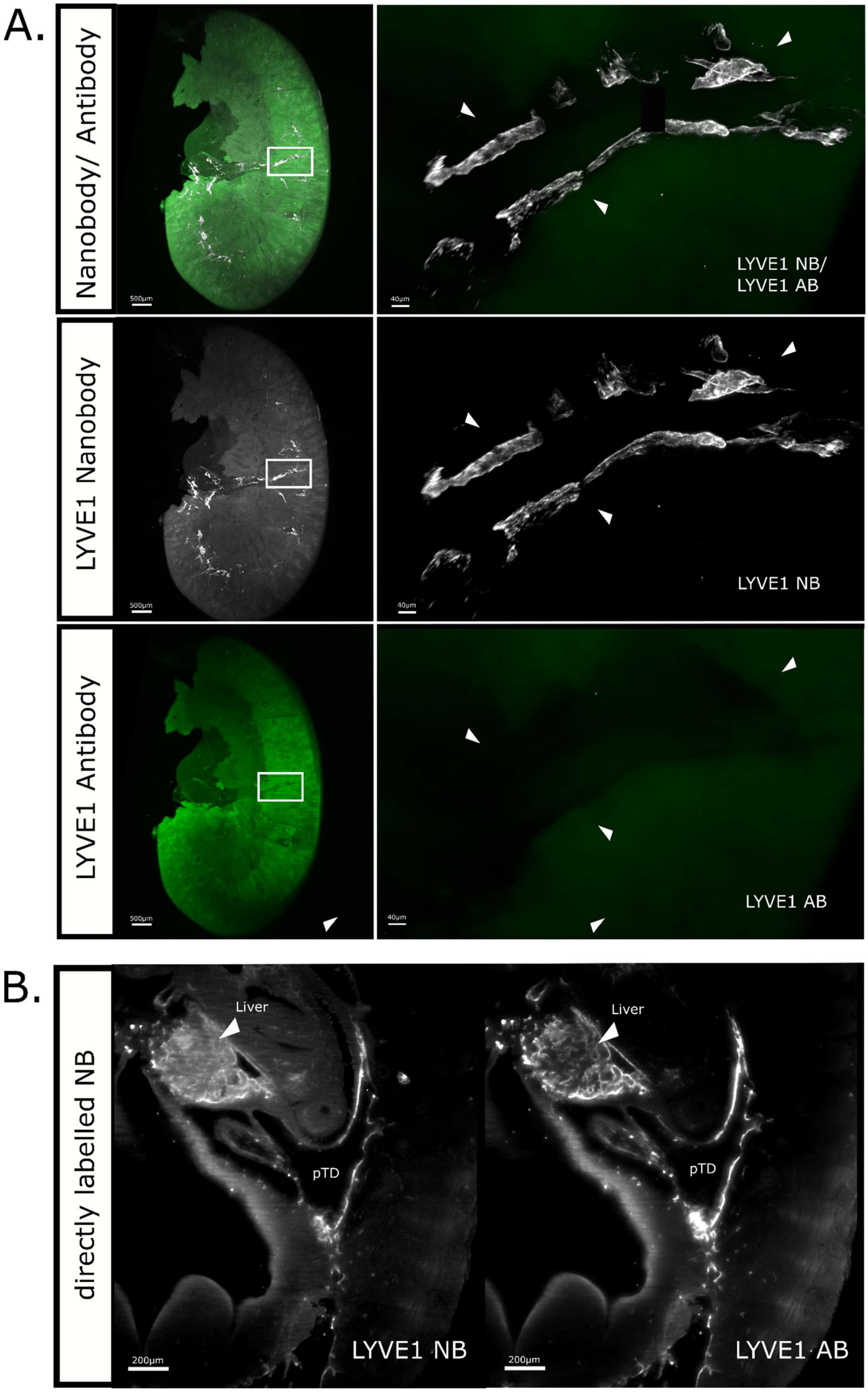
A.
Nanobody/ Antibody
LYVE1 NB/
LYVE1 AB
LYVE1 Nanobody
LYVE1 NB
LYVE1 Antibody
LYVE1 AB
500µm
40µm
B.
directly labelled NB
Liver
pTD
LYVE1 NB
LYVE1 AB
200µm

Supplementary Fig. 4 Effective visualisation of lymphatic vessels in reduced experimental time using anti-LYVE1 nanobodies.

(**A**) Mouse kidney sections (~2 mm) were stained for 4 hours with either anti-LYVE1 nanobodies or anti-LYVE1 IgG antibodies as primary antibodies. Fluorescence detection was performed overnight using either a AlexaFluor-labelled anti-His antibody (for nanobody detection) or an anti-goat AlexaFluor-labelled antibody (for IgG detection). While the anti-LYVE1 nanobodies successfully visualized lymphatic vessels within 4 hours of incubation (arrowheads), the anti-LYVE1 IgG antibodies failed to produce any staining, and no vascular structures were detected throughout the sample. Pearson correlation coefficient r=0. All images are Imaris rendered 3D reconstructions. The left presents overview images displaying a reconstruction of the entire kidney section, while the right shows a magnified view of the region marked with a square within the renal cortex in the overview. Scale bar: 500 µm/ 40µm. (**B**) Direct labelling of nanobodies using NHS ester in an E11.5 embryo. The primordial thoracic duct (pTD) is visualized in both channels. Pearson correlation coefficient r=0,94. All images shown are single z-slices. Scale bar is 200 µm.

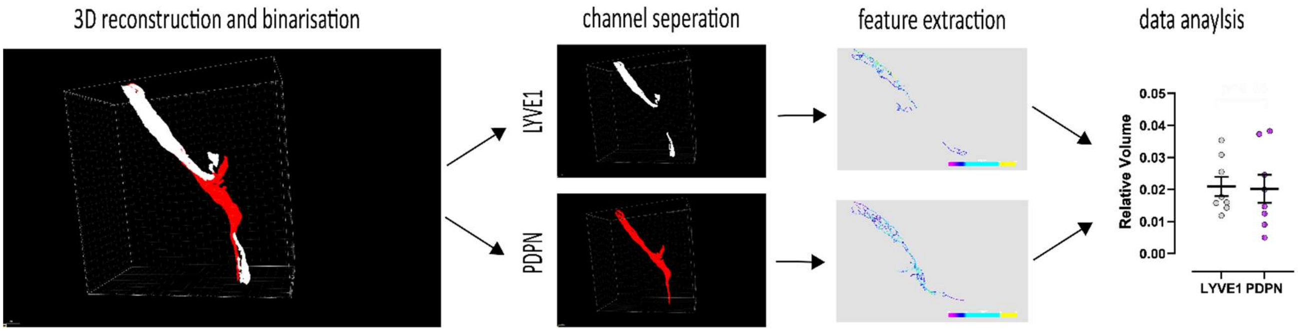

Supplementary Fig. 5 Overview of quantification process

Z-stack data was 3D reconstructed using Imaris software. The Imaris feature “Isosurface Render” enabled binarization of desired vessel structures. Thresholds were manually chosen and unwanted structures such as glomeruli in the PDPN channel were actively deleted within the surface feature. This was performed for both channels individually. Following the creation of two separate surfaces for the PDPN and LYVE1 channel, both channels were masked and separately saved as TIFF. Next, vessel volume was extracted using the open-source software VesselVio [1]. Lastly, values for separate channels of one sample were compared, visualised and statistical testing was carried out.

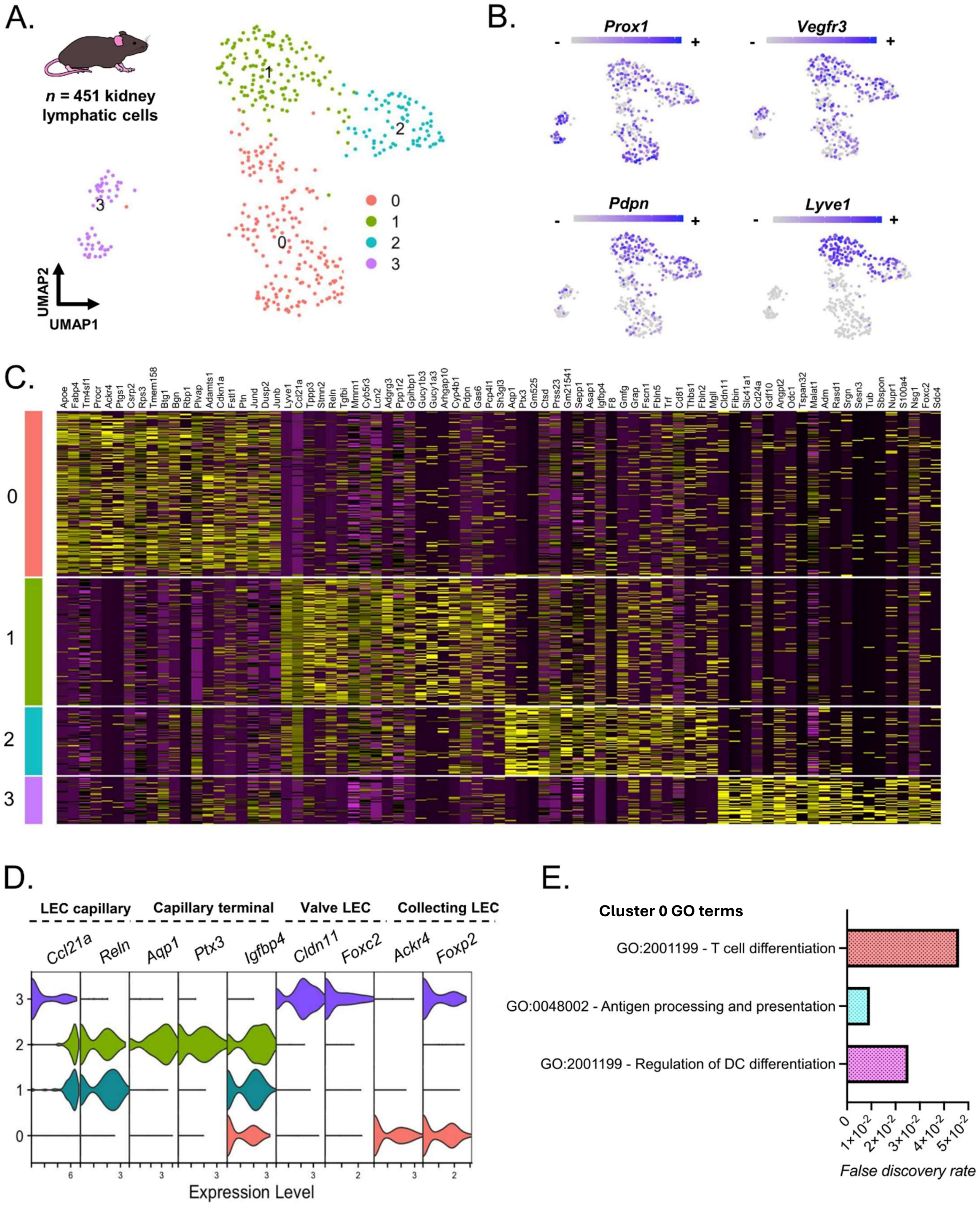

A.
n = 451 kidney lymphatic cells
UMAP2
UMAP1
0
1
2
3
B.
Prox1
Vegfr3
Pdpn
Lyve1
-
+
C.
Apoe Fabp4 Tm4sf1 Procr Ackr4 Ptgs1 Csrp2 Rps3 Tmem158 Btg1 Bgn Rbp1 Plvap Adamts1 Cdkn1a Fstl1 Ptn Jund Dusp2 Junb Lyve1 Ccl21a Tppp3 Stmn2 Reln Tgfbi Mmrn1 Cyb5r3 Lcn2 Adgrg3 Ppp1r2 Gpihbp1 Gucy1b3 Gucy1a3 Arhgap10 Cyp4b1 Pdpn Gas6 Pcp4l1 Sh3gl3 Aqp1 Ptx3 Gm525 Ctsd Prss23 Gm21541 Sepp1 Asap1 Igfbp4 F8 Gmfg Grap Fscn1 Fbln5 Trf Cd81 Thbs1 Fbln2 Mgll Cldn11 Fibin Slc41a1 Cd24a Gdf10 Angpt2 Odc1 Tspan32 Malat1 Adm Rasd1 Srgn Sesn3 Tub Sbspon Nupr1 S100a4 Nsg1 Foxc2 Sdc4
D.
LEC capillary
Capillary terminal
Valve LEC
Collecting LEC
Ccl21a
Reln
Aqp1
Ptx3
Igfbp4
Cldn11
Foxc2
Ackr4
Foxp2
Expression Level
E.
Cluster 0 GO terms
GO:2001199 - T cell differentiation
GO:0048002 - Antigen processing and presentation
GO:2001199 - Regulation of DC differentiation
0
1×10⁻²
2×10⁻²
3×10⁻²
4×10⁻²
5×10⁻²
False discovery rate

Supplementary Fig. 6 Characterisation of Lyve1+ and Lyve1- lymphatics in the mouse kidney using single-cell RNA sequencing

(**A**) Uniform manifold approximation and projection (UMAP) of single-cell RNA-sequencing (scRNA-seq) dataset of 12-week-old C57Bl/6 wildtype kidneys [2] from which 451 lymphatic cells were isolated. Unsupervised clustering resolved four transcriptionally distinct subclusters of kidney lymphatic endothelial cells. (**B)** Feature plots demonstrating the widespread expression of canonical lymphatic markers *Prox1*, *Vegfr3*, *Pdpn* throughout subclusters. Conversely *Lyve1* was enriched within clusters 1 and 2, with scant expression observed in clusters 0 and 3. (**C**) Heatmap of the top 20 differentially expressed genes (DEG) from each of the four clusters. (**D**) Violin plot showing selected DEGs corresponding to known markers of hierarchical anatomical identity within lymphatics. Capillary lymphatic markers (*Ccl21a* and *Reln*) were enriched in clusters 1 and 2. Capillary terminal lymphatic markers (*Aqp1*, *Ptx3* and *Igfbp4*) were enriched in cluster 2. Valve lymphatic cell markers (*Cldn11* and *Foxc2*) were enriched in cluster 3. Collecting lymphatic vessel markers (*Ackr4*, *Foxp2*) were enriched in cluster 0. (**E**) Gene ontology (GO) analysis of DEGs from cluster 0. Select GO terms are presented relating to immune modulation.

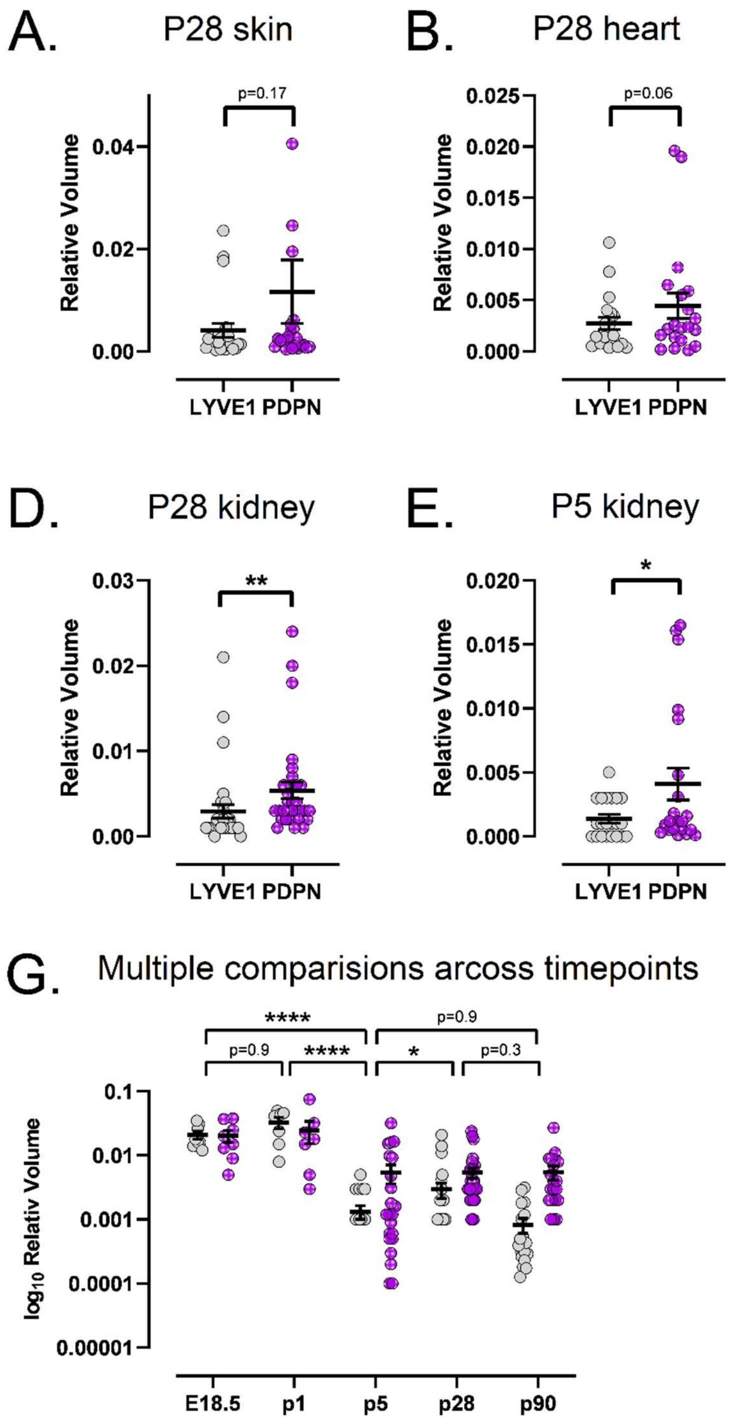

Supplementary Fig. 7. Quantitative analysis of LVYE1⁺ and PDPN⁺ volume per region of interest

Quantitative analysis of the relative volume of LYVE1+ and PDPN+ lymphatic vessels, with each data point representing one region of interest and error bars indicating the standard error of the mean. Vessel volumes were adjusted to the overall sample volume. Paired Student's t-

tests revealed no significant difference between the relative volumes of PDPN and LYVE1 in P28 skin (n=23, p=0.169, mean difference = 0.0075, t=1.1421, df=22, r =0.73 ± 0.11), heart (n=20, p=0.063 mean difference = 0.0017, t=1.976, df=19, r=0.67 ± 0.11), or lung (n=18, p=0.097, mean difference = 0.0007, t=1.765, df=16, 0.71 ± 0.14) (**A-C**). In P28 kidney (n=31, p=0.009; mean difference = 0.0025, t=2.753, df=30, 0.28 ± 0.09) (**D**), P5 kidney (n=21, p=0.014; mean difference = 0.0027, t=2.684, df=20, r = 0.33 ± 0.13) (**E**) and P90 kidney (n=19, p=0.004; mean difference = 0.0047, t=3.321, df=18, r = 0.30 ± 0.07) (**F**) a significant difference was found. E18.5 and P1 kidneys were imaged as a whole (see **Fig. 3B-C**). One-way ANOVA revealed a significant difference among organs for Pearson correlation coefficients (p < 0.0001), with kidney significantly lower than other organs (Tukey's post hoc test, p < 0.0001). (**G**) Statistical analysis of LYVE1 volume dynamics across five developmental timepoints using nonparametric multiple comparisons (estimation method = global pseudo ranks, type of contrast = Tukey, confidence level = 95 %) [3] revealed a significant overall alteration (overall p value=1.02 x 10^{-8}, quantile=2.75). No significant changes were found between E18.5 and P1 (p=0.96) or between P28 and P90 (p=0.34). Significant differences were observed between P1 and P5 (p=3.48e-07), P5 and P28 (p=0.038), E18.5 and P28 (p=1.02e-08), P1 and P28 (p=1.49e-06), and P1 and P90 (p=5.82e-05). No significant change was detected between P5 and P90 (p=0.9). This was supported by one-way ANOVA of Pearson correlation coefficients, which showed a significant overall decline across developmental timepoints (p < 0.0001), with post-hoc Tukey comparisons confirming a significant decrease from P1 to P5 (p < 0.0001).

Supplementary Video 1. Visualisation of P28 renal lymphatic vasculature by anti-LYVE1 nanobodies in comparison to anti-LYVE1 IgG antibodies

Video of 3D reconstruction of anti-mLYVE1 nanobodies (white) and anti-mLYVE1 IgG antibodies (green) visualisation of renal lymphatic vasculature. Superficial hilar lymphatics are visualised by anti-mLYVE1 Ab, whereas a vessel plexus further within the intact organ can only be detected by the anti-mLYVE1 nanobodies.

Supplementary Video 2. Visualisation of P28 lymphatic vasculature in murine lung lobe by anti-LYVE1 nanobodies in comparison to anti-LYVE1 IgG antibodies

Video of 3D reconstruction of anti-mLYVE1 nanobodies (white) and anti-mLYVE1 IgG antibodies (green) visualisation of lymphatic vasculature in a pulmonary lobe. The anti-mLYVE1 IgG antibody channel visualises singular lymphatic vessels within the lung with visible gaps in staining, whereas the anti-mLYVE1 nanobodies enable consistent vessel visualisation.

REFERENCES

1. Bumgarner, J.R. and R.J. Nelson, *Open-source analysis and visualization of segmented vasculature datasets with VesselVio.* Cell Rep Methods, 2022. **2**(4): p. 100189.
2. Subramanian, A., et al., *Obesity-instructed TREM2high macrophages identified by comparative analysis of diabetic mouse and human kidney at single cell resolution.* bioRxiv, 2021: p. 2021.05.30.446342.
3. Konietschke, F., et al., *nparcomp: An R Software Package for Nonparametric Multiple Comparisons and Simultaneous Confidence Intervals.* Journal of Statistical Software, 2015. **64**(9): p. 1 - 17.
